# Progress in Development of Photocatalytic Processes for Synthesis of Fuels and Organic Compounds under Outdoor Solar Light

**DOI:** 10.1021/acs.energyfuels.2c00178

**Published:** 2022-04-13

**Authors:** Alexey Galushchinskiy, Roberto González-Gómez, Kathryn McCarthy, Pau Farràs, Aleksandr Savateev

**Affiliations:** † Department of Colloid Chemistry, Max Planck Institute of Colloids and Interfaces, Am Mühlenberg 1, 14476 Potsdam, Germany; ‡ School of Chemistry, Ryan Institute, National University of Ireland, Galway H91 CF50, Ireland

## Abstract

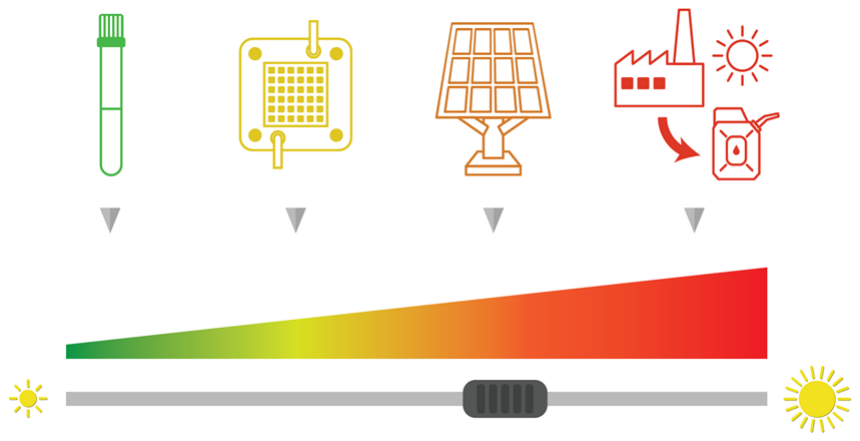

With photovoltaics becoming a mature, commercially feasible technology, society is willing to allocate resources for developing and deploying new technologies based on using solar light. Analysis of projects supported by the European Commission in the past decade indicates exponential growth of funding to photocatalytic (PC) and photoelectrocatalytic (PEC) technologies that aim either at technology readiness levels (TRLs) TRL 1–3 or TRL > 3, with more than 75 Mio€ allocated from the year 2019 onward. This review provides a summary of PC and PEC processes for the synthesis of bulk commodities such as solvents and fuels, as well as chemicals for niche applications. An overview of photoreactors for photocatalysis on a larger scale is provided. The review rounds off with the summary of reactions performed at lab scale under natural outdoor solar light to illustrate conceptual opportunities offered by solar-driven chemistry beyond the reduction of CO_2_ and water splitting. The authors offer their vision of the impact of this area of research on society and the economy.

## 1 Introduction

With the depletion of fossil fuel reserves,^1,2^ alternative sources, such as solar irradiation, grow more important not only for energy generation but also for the chemical industry, due to being essentially a cost-free and abundant power source to drive chemical transformations. A number of bulk products can be obtained by photocatalytic reaction under sunlight irradiation, such as hydrogen, syngas, methanol, formaldehyde, and formic acid.^3,4^ Nonbulk chemicals, such as pharmaceuticals, additives, and reagents, can also be obtained by means of sunlight irradiation, although the demand and economic impact for this kind of solar chemistry is much less.

Another important factor influencing the development of solar photocatalysis is the accumulation of carbon dioxide, which contributes more and more significantly to the overall carbon mass in the Earth’s atmosphere. With a current average concentration in the air between 400 and 500 ppm and an overall atmospheric mass of around 3200 gigatons,^5^ CO_2_ represents a growing threat to the biosphere but also an attractive carbon reservoir. Photocatalytic fixation of carbon dioxide in the form of bulk materials and solvents may contribute to the solution of this problem by decreasing the rate of production for corresponding petrochemical products, reducing the overall negative environmental impact of oil industry, and lowering atmospheric CO_2_ concentration at the same time, once an efficient direct air capture (DAC) technology is developed.

Another alternative carbon source is biomass—around 146 billion metric tons are produced by plants each year,^6^ making it a widely available resource lower in price than fossil fuels and commercial carbon dioxide (liquefied CO_2_ and dry ice). It also has some additional key advantages; much like products of the oil industry, it can be stored for a prolonged amount of time and provide a broad scope of chemical products.^7^ Fractionation of otherwise non-processable biomass, such as wastes of agriculture and wood processing, provides several products from cellulose and lignin decay (see Section 4.2.2 of the review for further discussion). These products can be utilized on their own as ready-to-use chemicals or may be converted into biocompatible and biodegradable polymers.

This review is focused on chemical, engineering, and economic aspects of bulk solar photocatalysis, i.e., CO_2_ and biomass processing, including an overview of novel larger-scale experimental and pilot-scale reactors reported in the past several years. Some proof-of-concept reports, namely, complex organic transformations under sunlight and experimental photocatalytic setups, are also covered with emphasis on more recent results and precious metal-free catalysts. The aim of this work is to cover *exclusively natural sunlight-driven* photocatalytic processes as a demonstration of researchers’ intents for potential industrial applications, and thus, examples under simulated or artificial light use are omitted.

## 2 Impact of Photocatalysis under Outdoor Solar Light on Economy

One of the main arguments for deploying technologies based on harvesting and utilization of solar light is the amount of energy that Earth’s surface receives from the sun, which exceeds the annual demand of the entire population.^8^ However, the geographic location and portfolio of chemical goods generated with the aid of solar light will define the overall economic viability of a certain technology. Solar irradiance is the highest in the tropical region but decreases when moving north or south from the equator.^9^ Therefore, solar-driven technology will have the highest societal and economic impact in the regions where solar irradiance is the strongest and available throughout the year.

Due to the limited permittivity of electromagnetic radiation into the bulk of a photoreactor (or photoelectrode), as inferred from the Beer–Lambert law, the productivity of photo(electro)chemical reactors scales with their surface area rather than volume. Different geographic locations have distinct economic potential defined by the level of their development. For example, to be economically competitive, 1 m^2^ of land in a large urban center must generate goods and services with a higher value compared to 1 m^2^ in a rural area. Taking into account these two facts, bulk commodities and solar fuels, which are at the origin of the value chain rather than end products, can succeed only in areas where solar irradiance is high and land is available. On the other hand, fine chemicals with much higher market prices are more competitive in the regions with a higher cost of land and more diluted solar radiation.

Deploying solar panels and other kinds of solar harvesting devices might have an environmental impact on the local ecosystem, considering that these are manufactured objects.^10^ As solar-driven chemistry attracts more and more attention, consideration goes beyond only scientific optimization of the photocatalyst and photoreactor; those focused on economic viability are necessary to develop and deploy the right technology at the right place.

## 3 Advantages and Limitations of Photocatalysis under Outdoor Solar Light

The main advantage of solar photocatalysis is, undoubtedly, an abundance of a free energy source required to drive the reaction, which now can be effectively seen as a regular ambient temperature reaction in terms of energy balance. Moreover, with precise calculation of light concentration and dissipation of heat, a desired internal temperature can be achieved without utilizing external heating devices. However, using a convenient natural source has its own limitations, different from those of commercial light sources and solar simulators. For instance, solar irradiation spectrum intensity is affected significantly by light absorption of gases in the atmosphere (N_2_, O_2_, CO_2_, Ar, O_3_, NO_*x*_, SO_2_, CH_4_) and air humidity; aerosol particles also take part in the overall process, diffusing incoming irradiation.^11^ The uneven distribution of irradiation intensity onto the spectral range is also to be taken into account; most of the output is spread across the visible and NIR regions with little impact on UV region, directly affecting reaction design. If a UV photochemical or photocatalytic reaction is desirable, the primary concern is the reactor materials, and among a whole variety of glasses and plastics, there are only a few which are both transparent below the 300 nm threshold and stable enough to operate under outdoor conditions. Highly fluorinated polymers are particularly good for this purpose, allowing for complex shapes of absorbing windows; however, they have poor mechanical characteristics for increased pressure and a flow working regime. Thus, wall thickness has to be increased, leading to absorption losses.^12^ On the other hand, low-iron silica-doped borosilicate glasses have excellent resistance to outdoor weather conditions and transparency until 280–285 nm, but they suffer from “UV solarization”, a process attributed to changes in the material structure under prolonged high-energy irradiation, in particular, the oxidation of Fe^2+^ to strongly absorbing Fe^3+^.^13^ However, increasing use of photoredox catalysts in the past decade for various kinds of reactions can assist in neglecting these strict requirements by shifting the operational range to visible light, which is suitable for a broad selection of transparent materials.

Additionally, there is a couple of more obvious parameters related to irradiation, namely, the diurnal light cycle in the operating area and weather conditions. Effectively, these make subtropical and tropical countries seem to be the most suitable areas for solar chemical plants due to optimal insolation, day–night time ratio, and lack of cloudy days, as cloudiness can obscure up to 50% of solar irradiance.^14^

Other factors to consider are chemical kinetics and thermodynamics, along with flow dynamics and mass transfer. Scaling up photochemical reactions is much harder than conventional ones because of the surface-dependent character of the overall process – light cannot effectively penetrate into the bulk of concentrated reaction medium. The surface-to-volume ratio changes in reverse proportion to the linear size increase, thus rendering batch reactors completely ineffective for large-scale applications. Furthermore, employing heterogeneous photocatalysts provides additional catalysts–reagents phase interactions that should also be taken into consideration, as well as some specific problems like fouling (adhesion of photocatalyst to the reactor surface due to photocorrosion, surface potential redistribution, or adsorption of tarry byproducts).^15^ Therefore, an approach to reactor design changes drastically as new parameters are accounted for.

Several metrics to compare photocatalytic activity exist, and the ones most frequently used are apparent quantum efficiency (AQE), also noted as apparent quantum yield (AQY) of the catalyst, and external quantum efficiency (EQE). AQE refers to the ratio of reacted molecules to the amount of incident photons under monochromatic irradiation; meantime, EQE reflects a spectral irradiation and is a more representative value for solar photocatalysis. However, occasionally, these values are used interchangeably.^16^ EQE values of current photocatalysts typically do not exceed 30% (visible light wavelengths ≤ 500 nm are usually used as a reference),^17−23^ but there are a number of recent papers reporting promisingly high (>60%) efficiencies for hydrogen evolution.^24−26^

## 4 Operating and Developing Technologies

### 4.1 Funding Statistics: From Basic Research to Applied Technology

The European Commission (EC), as the major research funding organism in Europe, promotes innovative green projects to decarbonize the European economy. The research and innovation projects stretch from basic principles to technology validation, to pushing them further toward industrial-scale processes, and finally, to their commercialization. In alignment with the EC goals, solar-driven technologies, such as photoelectrocatalysis (PEC) and photocatalysis (PC), have become more relevant in recent years due to their proven applications in the production of value-added chemicals generated by sunlight (solar fuels and solar raw chemicals).

**Figure 1:**
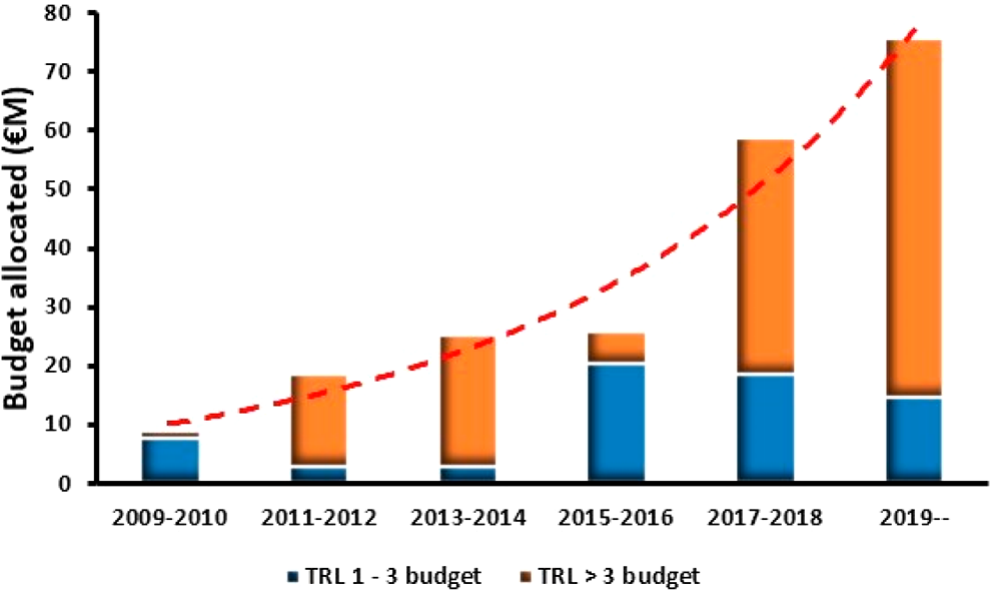
Total budget allocated biannually for PEC- and PC-based projects in Europe bundled according to the TRL scale. Exponential line was fitted to the total budget awarded. Data on funded projects have been taken from CORDIS.

The increasing relevance of solar-driven technologies can be monitored by the EC budget allocated per year for PEC- and PC-based projects. In the past decade, the number of projects awarded by the EC has significantly increased, and more notably, the budgets allocated. About 10 years ago (2009–2010), less than €10 M was assigned to PEC- and PC-based projects compared to the more than €75 M designated from 2019 onward; the exponential growth of the total budget allocated biannually is highlighted in Figure 1. With the launch of the EU Green Deal, this figure is expected to increase even more, as a surge in projects directed at innovating green technologies will need to be financed by European agencies. Regarding technological development, early in the past decade, awarded solar-driven projects were mainly based on investigating basic principles of PEC and PC technologies at low technology readiness levels (TRLs). Meanwhile, due to the scarcity of proven systems to be validated, TRL > 3 projects were not available as funding calls focused on alternative green technologies, such as electrolysis. In later years, PEC- and PC-based projects that go beyond the proof-of-concept (higher TRL) have been prioritized and are seeing a sharp increase in the funding. On the other hand, TRL 1–3 projects have suffered lesser growth recently.

In the early 2010s, PEC- and PC-based projects funded by the EC focused on producing solar fuels, namely, water splitting, to generate hydrogen fuel—a simpler application of this technology in comparison with the production of solar fuels from CO_2_. However, the need of producing carbon-based materials for the chemical industry has shifted projects toward using CO_2_ as a starting molecule. Recently, solar-driven awarded projects have centered their attention on the production of solar chemicals, such as short-chain alcohols and carbon-based precursors for manufacturing value-added chemicals. In terms of solar-driven technologies, early in the past decade, PEC- and PC-based projects were comparably awarded. Despite the fact that the number of financed projects has steadily increased throughout the decade, PEC-based funded projects have seen a slight decline over the last five years as the focus turns toward PC-based projects, which have tripled. The discussed trends and a compilation of the PEC- and PC-based projects funded by the EC since 2009 is presented in Table S1 in the SI.

Throughout the decade, the attention was first placed on researching basic principles of PEC- and PC-based technologies, with limited room for validating the concepts. *PECDEMO*, which started back in 2014, was one of the first TRL > 3 projects using PEC-based technology for hydrogen fuel production, aiming to build a hybrid PEC–photovoltaic device capable of splitting water in a solar-driven process. *PECDEMO* worked with industry collaborators to scale-up these devices; however, the solar-to-hydrogen (STH) conversion efficiencies at large-scale were not good enough for the devices to be commercially viable. Since then, more projects have been awarded in order to scale up solar-driven processes and validate them. In 2019, *Bac-to-Fuel* began working on the conversion of CO_2_ and H_2_ into biofuels. They aim to produce renewable hydrogen from the photocatalytic splitting of water. The produced green hydrogen is then combined with CO_2_ to produce cost-effective biofuels using enhanced bacterial media in an electrobiocatalytic cell. The built prototype will be validated to TRL 5.

Recently, with the aim to produce green raw materials for the chemical industry, elaborate carbon-based molecules have been the goal of PEC- and PC-based projects. Ethylene, one such chemical of interest, has been the target molecule of two recently TRL > 3 EU-funded projects. *FlowPhotoChem*, a project that commenced in June 2020, aims to construct an integrated modular system consisting of three different reactors (PEC, PC, and electrochemical reactors), which will work in flow to produce ethylene by means of CO_2_ reduction, as well as other value-added chemicals, namely, ethanol, ethyl acetate, and *n*-propanol. Similarly, *Sun2Chem*, which began in October 2020, intends to produce ethylene from CO_2_ using a tandem PEC device and a PC reactor. Furthermore, other carbon-based chemical targets have been the focus of TRL > 3 projects aiming to go beyond the proof-of-concept of these technologies and scale up the production of green chemicals by using PEC- and PC-based technologies. The *DECADE* project, which began in May 2020, focuses on using a novel PEC system to produce green solvents, ethyl acetate and ethyl formate, from waste CO_2_ and bioethanol. This system will be scaled up, and a prototype will be designed, manufactured, and validated to TRL 5. Finally, another project that was launched in May 2020, *SunCoChem*, seeks to produce valuable oxygen-containing green chemicals using a PEC tandem reactor by reacting CO_2_ and H_2_O with olefins, such as butene and limonene.

As Europe moves toward a decarbonized economy, there is the need for an increased allocation of funding toward the application of green technologies, like PEC and PC. In the short term, it is expected that solar-driven technologies will be scaled up and validated, so that they can be industrialized in the midterm and even compete at a commercial level. Moreover, following the example of the United States and Japan, the public–private initiative SUNERGY,^27^ created from the coordination support actions ENERGY-X and SUNRISE, together with the EC, are in the process of finalizing a European roadmap that will be the base for future funding calls. At a global level, Mission Innovation 5 on converting sunlight already published a roadmap on February 2021 with the objective to link these innovative technologies with a green circular economy.^28^

### 4.2 Main Chemical Targets

#### 4.2.1 CO_2_ Reduction Products

Carbon dioxide is the primary source material for organic solar fuel research due to the fact that this is a common and readily available carbon-based compound, which has a growing tendency to accumulate in the Earth’s atmosphere.^29^ Therefore, decarbonization becomes one of the main tasks of solar economy.

**Scheme 1:**
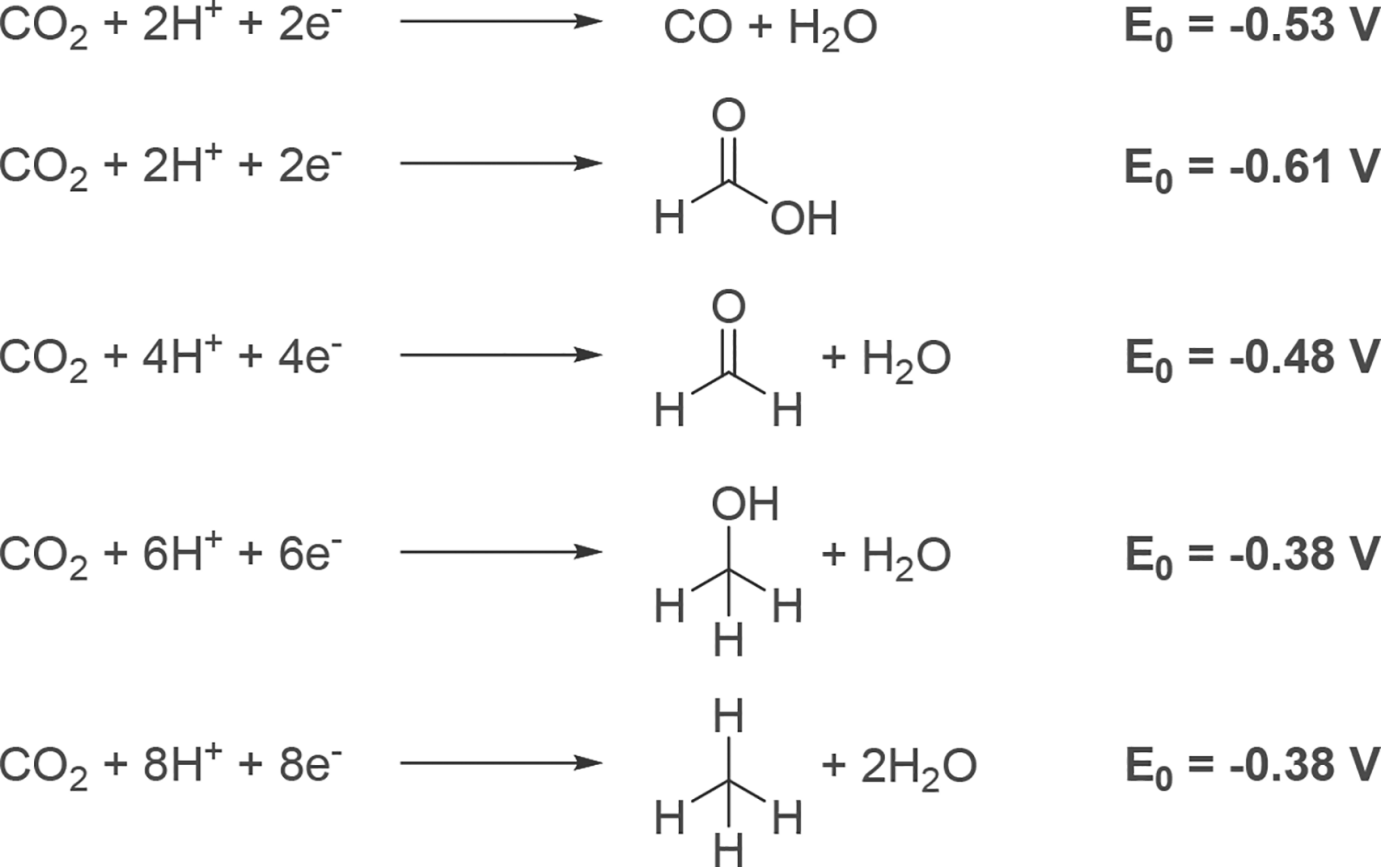
Reactions of CO_2_ Reductions and Their Electrochemical Potentials Adapted with permission from ref (30). Copyright 2009, Royal Society of Chemistry.

At the present moment, there are several approaches for chemical utilization of atmospheric carbon dioxide relying on different electrochemical reactions^30^ (Scheme 1).

As seen from this scheme, there are multiple opportunities for proton-coupled electron transfer (PCET) processes leading to methane (full reduction), methanol, formaldehyde, and acetic acid (partial reductions); however, they require multiple electron transfer reactions to proceed and thus demand catalyst design to be efficient and selective toward a certain product. Reduction without C–H bond formation produces carbon monoxide, which could be then mixed with hydrogen on site to form syngas, a valuable precursor for numerous bulk chemicals^31^ which is generally obtained otherwise by coal gasification or methane steam reforming.^32^ Additionally, there is a competitive process of hydrogen evolution typically accompanying CO_2_ reduction at lower pH values, interfering with the main reaction and decreasing the catalyst efficiency.^33,34^ Finally, the structure and morphology of a catalyst itself are other determining factors for selectivity, as CO_2_ and water need to be adsorbed first on its surface prior to be photoactivated, and distribution of local charges and vacancies is crucial in this matter.^35^ Common heterogeneous catalysts currently studied for carbon dioxide photoreduction include metal (primarily copper) oxides and chalcogenides systems,^36,37^ carbon nitrides,^38^ graphene materials,^39^ MOFs,^40^ and MXene-based cocatalytic tandems.^41^

**Figure 2:**
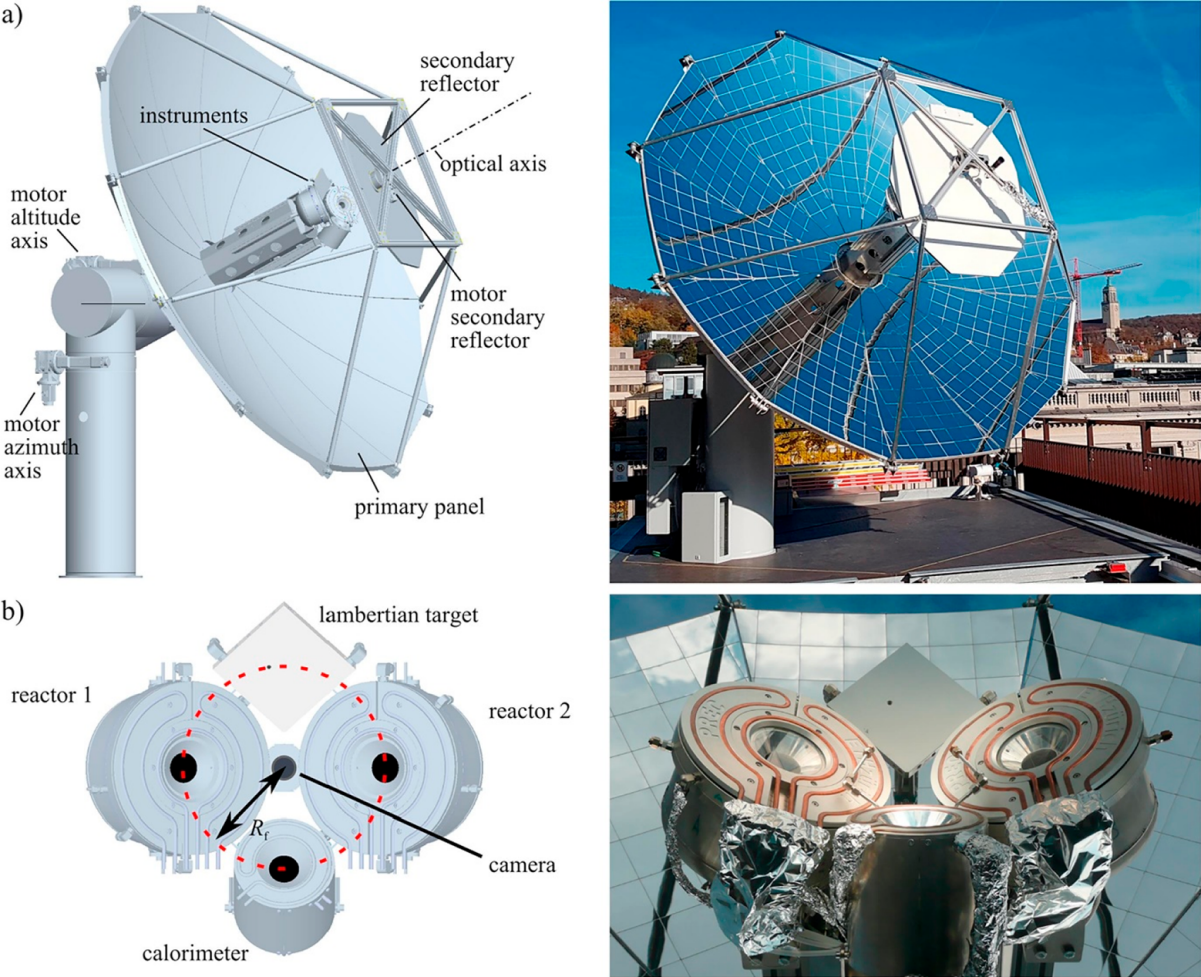
Rendered schematics and photos of (a) solar tracking parabolic concentrator and (b) mounted solar reactors for photothermal CO_2_ reduction in ETHZ. Reprinted with permission from ref (44). Copyright 2018, Elsevier.

**Figure 3:**
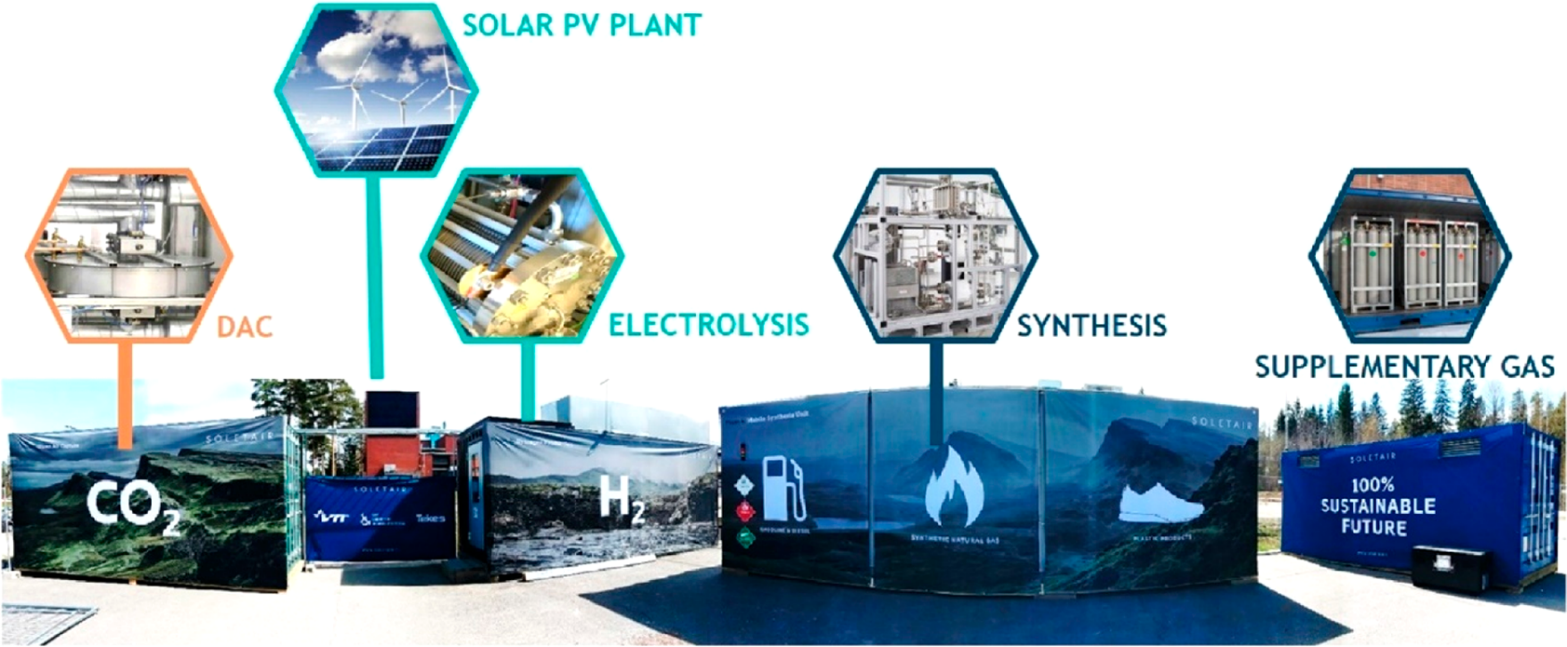
Outer view of SOLETAIR CO_2_ processing setup modules. Reprinted with permission from ref (45). Copyright 2018, Elsevier.

Recent studies^42^ propose a key role of solar energetics in solving the CO_2_-fixation problem by converting it into methanol or longer atom chain carbohydrates, such as Fischer–Tropsch fuels (directly as C2+ fraction or via syngas) and polymers. Moreover, several later pilot plant projects relying on DAC technology demonstrate that there is a significant demand for solar fuels in the market; nonetheless, they do not employ photo(electro)chemical processes: the future Synhelion plant in Jülich, Germany, will utilize a solar concentrating thermal setup designed in ETH Zürich (Figure 2) with a solar-to-work efficiency of 67.3%,^43,44^ while a pilot SOLETAIR setup (Figure 3) in Lappeenranta University of Technology can simultaneously reduce CO_2_ and electrolyze water being driven by photovoltaic power with syngas cofeeding.^45^ So far, it is safe to conclude that the main bottleneck of the industrial photo(electro)chemical approach is the absence of efficient commercial photocatalysts and reactor designs that can handle this process at an economically feasible rate in comparison to the established process of photothermal reduction.^46^ Currently, there are several research initiatives working on resolving this particular problem.^47,48^ An additional issue is that a series of CO_2_ reduction products, mainly methanol and syngas (as an intermediate for C2+ products), possess significant market value, and their formation processes are competitive and rely on multiple factors,^49,50^ which imply division of selective transformations with differently designed catalysts for each setup, depending on the desired process, rather than formation of a mixture of valuable products with further separation. Another perspective industrial CO_2_-harvesting approach is the exploiting of the natural mechanism of photosynthesis in microalgae, although current technologies yield biodiesels too costly to be competitive on the market.^51^

**Figure 4:**
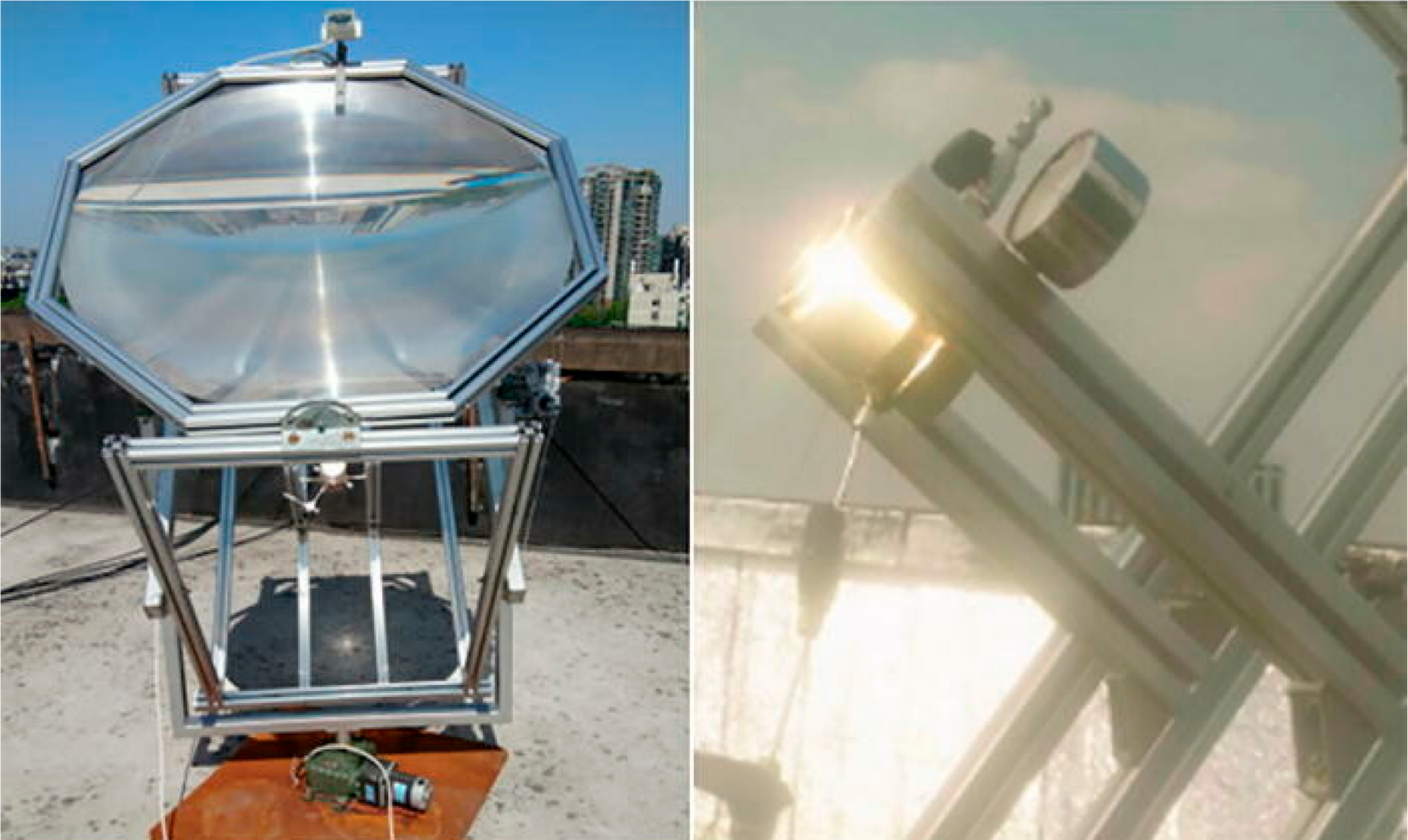
Solar concentrator setup for batch photocatalytic CO_2_ reduction (left) and close up of its reaction chamber (right). Reprinted with permission from ref (52). Copyright 2021. AIP Publishing.

In the literature, examples of natural sunlight utilization for CO_2_ reduction are quite scarce. Testing under standardized AM1.5 solar simulators is usually the option of choice since they allow the researchers to get more accurate and reproducible results for the scientific community. Nonetheless, for designing genuine pilot setups, tests under actual sunlight are crucial for obtaining information on productivity in their working conditions. In Table 1, selected reports on CO_2_ reduction under sunlight are summarized. In these works, the reaction is performed on various types of heterogeneous catalysts, including titania,^52,53^ graphitic carbon nitride (g-CN),^54^ metal–organic frameworks (MOFs),^55,56^ and metal phosphides;^57^ the research is focused on selectively yielding either syngas or methanol. There is, however, a notable example of obtaining a mixed C2 gas fraction from carbon dioxide and water on a pilot solar concentrator setup^52^ (Figure 4), giving acetylene and ethylene as major products with small methane impurity. The batch reactor chamber with a transparent window is mounted in the focus of a round parabolic mirror, allowing for concentration rates up to 800, according to the authors. Despite the current low conversion, with a batch reactor scheme and production rates lower than 1 mmol g^–1^ h^–1^ for individual compounds, this approach may have the potential to emerge actual C2+ solar fuel technology.

**Table 1:** Examples of Sunlight-Driven Photocatalytic CO_2_ Reduction in Literature

Entry	Catalyst	Catalyst efficiency (%)	Solvent	Conditions	Product(s)	Conv. (%)	Yield	ref
1	TiO_2_ nanotube arrays	STC 0.0025–0.012 (AM1.5)	H_2_O	Solar concentrator reactor with CR = 200–800, 0.05 MPa CO_2_ partial pressure, 3.5 h	CH_4_, C_2_H_2_, C_2_H_4_, C_2_H_6_	0.27	258, 3077, 1736, 929 μmol g^–1^, respectively (after 3.5 h)	(52)
2	Cu/C-codoped TiO_2_ nanoparticles	N/R	Seawater	3 wt % catalyst, constant saturation of solution with CO_2_	Methanol	N/R	188 μmol g^–1^ h^–1^	(53)
3	g-CN	AQE 2.4 (355 nm)	H_2_O	1 g L^**–**1^ catalyst, 3.4 atm CO_2_	Methanol	N/R	130 μmol g^**–**1^ h^**–**1^	(54)
4	Hf_12_-Ru-Re (2D-MOF)	N/R	CH_3_CN	0.1 μM cat. (rebased), 1 atm CO_2_, 24 h, 0.05 v/v TEOA, 0.1 M sacrificial agent	CO (HCO_2_H as a side product)	N/R (TON = 3596, 24 h)	N/R (selectivity for CO > 99%)	(55)
5	Ru@Cu-HHTP (Ru-sensitized MOF)	N/R	CH_3_CN/H_2_O 4:1	0.043 g L^**–**1^ catalyst, 1 atm CO_2_, 24 h, 0.3 M TEOA	CO	N/R	69.5 mmol g^–1^ h^–1^, selectivity 91.3%	(56)
6	Cd_4_P_2_Br_3_/Ni_*x*_P_*y*_	AQE 4.11 (artificial light), 9.83 (sunlight)	H_2_O	0.18 M Na_2_S, 0.24 M Na_2_SO_3_	CO, CH_4_, H_2_	N/R	9258 μmol h^–1^ g^–1^ (measured for H_2_)	(57)

#### 4.2.2 Biomass Valorization Products

Unlike readily available small molecules, biomass and its wastes are composed of various kinds of biopolymers (mostly lignin and cellulose). This feedstock provides a challenging target for selective reforming through a chemical pathway since they have no strict composition, and therefore, biotechnology seems a more efficient solution. Numerous photocatalytic approaches exist, although overall TRL for these transformations is still low, and pyrolysis and bioprocessing reactors could stay predominant for a while.^58−60^

**Scheme 2:**
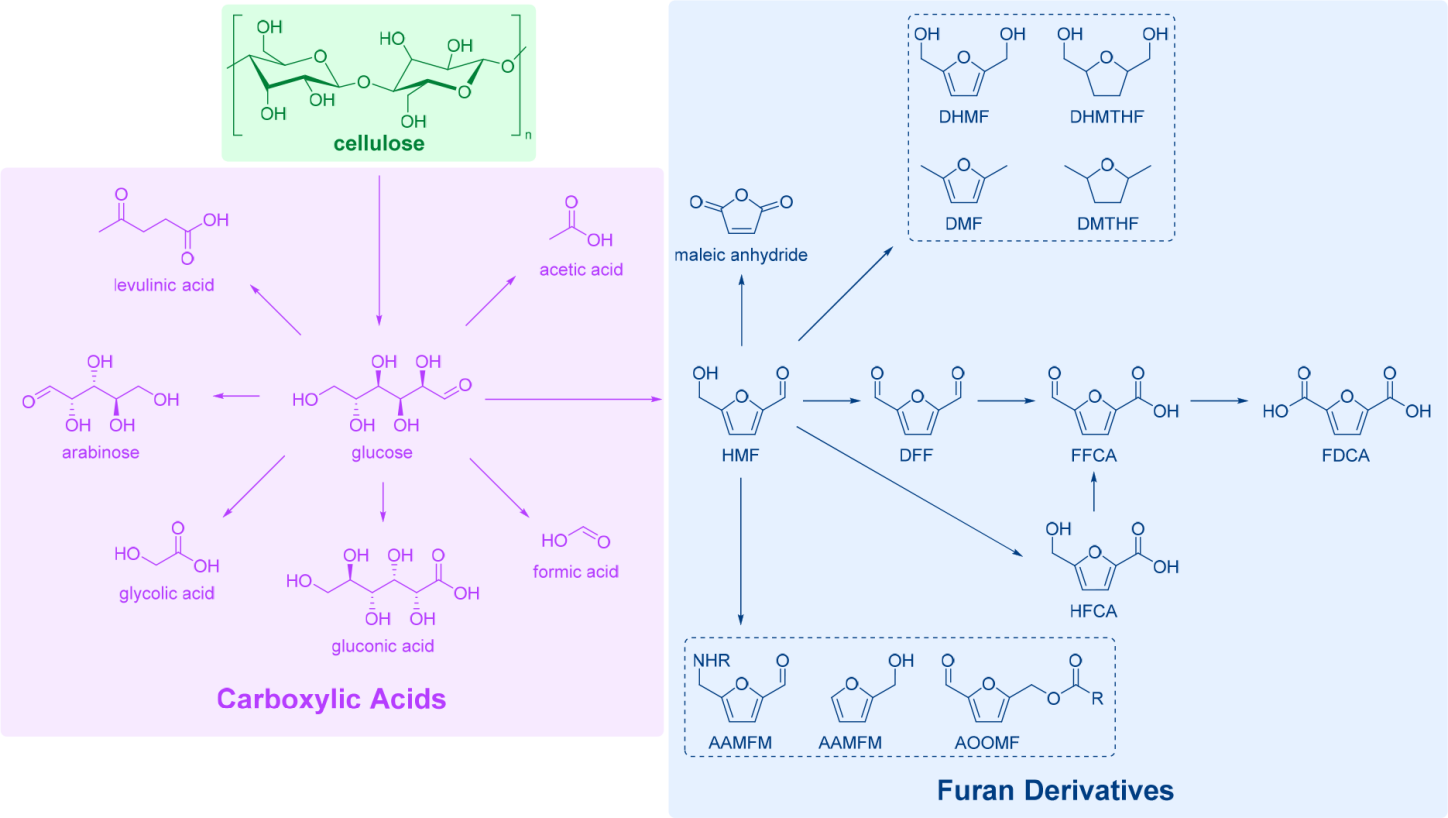
Pathways of Cellulose Biomass Processing. Adapted with permission from ref (61). Copyright 2021, Frontiers

The key transformations of cellulose biomass are shown on Scheme 2. First, the polymer chain is broken during hydrolytic pretreatment into glucose monomers, which can then be oxidized to lower-chain acids, such as acetic, formic, or glycolic acids; alternatively, chain shortening, terminal carbon oxidations, or rearrangement products are possible, including 5-hydroxymethylfurfural (HMF). The latter is another valuable precursor to a number of furan-based building blocks, as demonstrated in Scheme 2.^61^

To this day, there are few examples of photocatalytic biomass and biomass-derived molecules processing performed under actual solar irradiation, and the research is mostly centered around biomass-to-hydrogen conversion and transformation to small furan molecules.

Cellulose biomass (i.e., rice husk) was utilized as a sacrificial electron donor for hydrogen evolution accompanied by humins formation in the presence of a Pt/TiO_2_ photocatalyst. The yield of hydrogen varies from 4 to 8 μmol h^–1^ with 2 g L^–1^ catalyst loading; humin yields are not specified.^62^

Diformylfuran (DFF), an important monomer and a small molecules precursor, was prepared by Marcì et al., from HMF using thermally etched polymeric carbon nitride (CN) as a photocatalyst; the product was obtained with 20% yield and 88% selectivity after 4 h in the case of a catalyst adduct with hydrogen peroxide, while CN alone produced DFF with 47% yield and 38% selectivity.^63^ Another publication from Marcì et al. reports improved conversion rates up to 73% over porphyrin-impregnated CN under similar conditions; however, the selectivity for DFF dropped to 37%.^64^

Degradation of lignin is another attractive process for researchers as it allows access to several aromatic ring-containing products. For instance, 95% depolymerization in dioxane in 60–90 min was achieved with soft-template Zn_0.95_Bi_0.05_O nanocomposites obtaining various products, such as phenol (21%), 2-methoxy-4-methylphenol (16%), syringaldehyde, sinapyl alcohol, phthalates, and 4-hydroxy-benzoic acid.^65^

Recent results demonstrate both biomass processing and CO_2_ reduction combined in a single system using a cobalt(II) terpyridine catalyst immobilized on titanium dioxide. Carbon dioxide was reduced to CO, and pretreated cellulose was converted to formate with syngas: HCO_2_H ratio close to 1 and up to 39% yield based on cellulose. However, this reaction was only tested under a solar simulator.^66^

## 5 Proof-of-Concept Research on Organic Small Molecules

Since the 1990s, a significant amount of research was put into the photocatalytic or photochemical synthesis of valuable organic compounds, such as pharmaceutical building blocks and even final target molecules, under direct solar irradiation.^67^ Despite the efforts, the overall strategy seems less attractive in terms of economic feasibility than bulk small molecules or hydrogen due to a number of factors. First, the demand for these products is not nearly as high as for CO_2_ reduction or water splitting.^68^ Second, the insufficient TRL of pilot setups delays their implementation into the actual industrial production pipeline, hence the inability to provide enough experimental data for scaling up. Finally, there is a problem of reactor versatility for the processes that impose different parameters, such as light concentration factor, operational wavelength window (some reactions may require special optical filters or photon up-converters for UV or high-energy visible range), and compatibility of multiple phases in a concerted action. For instance, the setups for homogeneous monophasic processes and heterogeneous catalyst-mediated aerobic oxidation would change drastically due to the necessity to control dispersibility of the catalyst, gas–liquid mixing, flow mode, and more.^69^ Therefore, this section only covers a few selected reactions of potential value that have been tested under actual solar irradiation on a mmol scale in conventional lab equipment and cannot be considered as a technology ready for implementation; its purpose is to demonstrate the potential of a solar-to-chemical photocatalysis concept for future applications. The focus is set on net-oxidative and redox-neutral photocatalytic reactions, as they appeal most to the principles of green chemistry, meaning no costly sacrificial electron donors or acceptors are necessary for the process to occur.

### 5.1 Net-Oxidative Reactions

In net-oxidative photocatalytic reactions, the redox cycle usually serves to either implement oxygen atoms into a molecule (“oxygenase” type reactions) or to abstract protons from it, introducing new bonds (“oxidaze” type reactions).^70^ Photocatalytic oxidation is attracting researchers’ attention as it allows one to use atmospheric oxygen directly as a terminal oxidant, essentially rendering the whole process “green” and more cost -efficient.

**Scheme 3:**
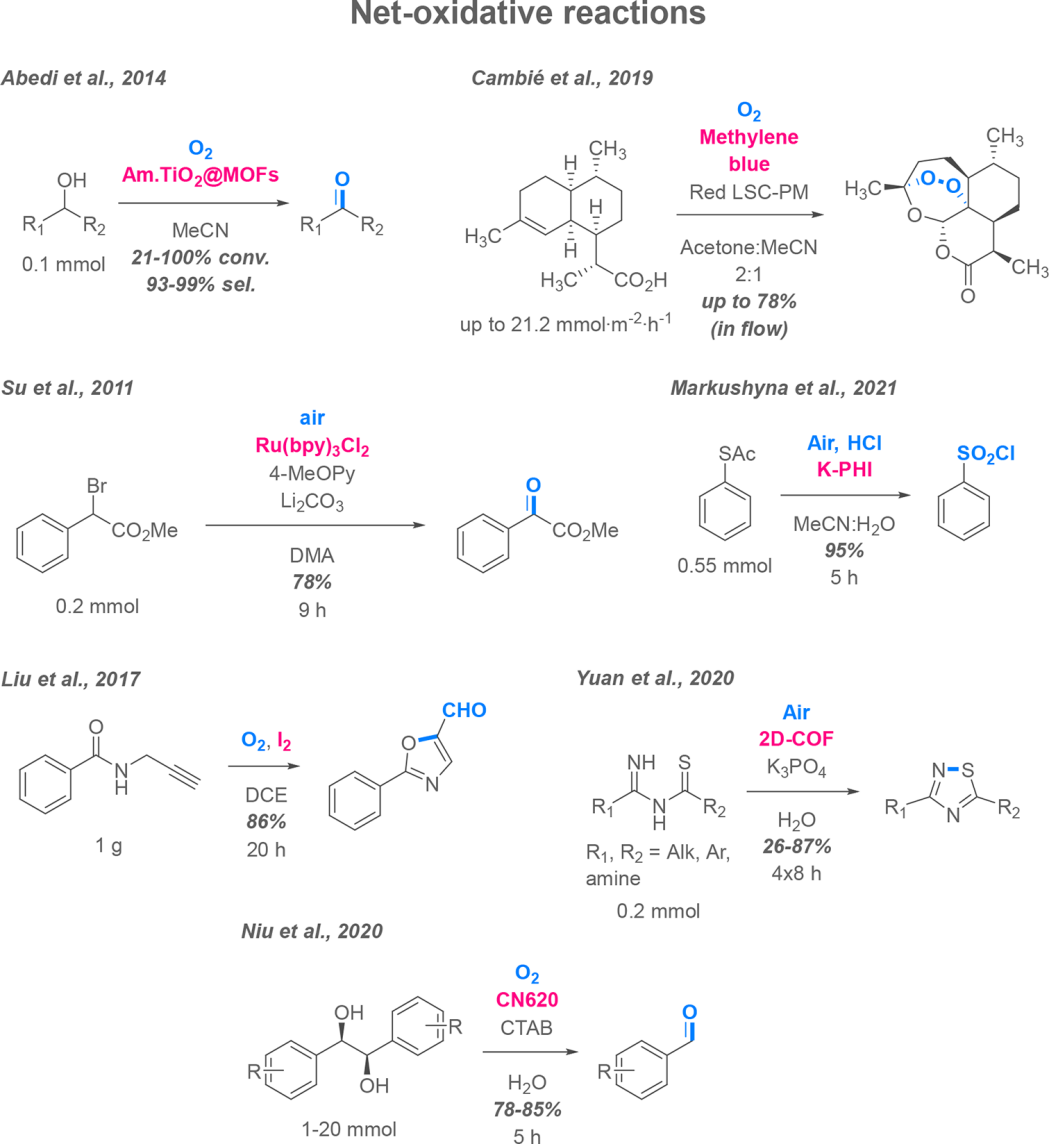
Proof-of-Concept Sunlight-Driven Net-Oxidative Photocatalytic Reactions Used in Organic Synthesis

Oxidation of alcohols, especially primary ones, is one of the most desired applications for organic photoredox catalysis due to the fact that it conventionally requires toxic and/or relatively expensive reagents to be selective toward carbonyl products, such as pyridinium chlorochromate (PCC) and Dess–Martin periodinane (DMP).^71,72^ Otherwise, multistep procedures are employed, including Swern-type DMSO-mediated oxidation reactions.^73^ These methods are often necessary to prevent overoxidation products when targeting aldehyde formation. Therefore, obtaining aldehydes and ketones by aerobic oxidation appears attractive in terms of cost efficiency and atom economy, resulting in the production of benzaldehydes from corresponding alcohols becoming one of the most popular “benchmark reactions” for testing novel photocatalysts. For example, solar oxidation of some benzyl alcohols was performed using MOFs as catalysts (Scheme 3).^74^

The second most prominent process for solar organic synthesis is endoperoxide formation. Artemisinins are a class of semisynthetic polycyclic endoperoxides that have been on the frontline of antimalarial therapy for the last decades.^75^ Despite the developing resistance to this type of drug, artemisinin and its derivatives are still in high demand on the market. While the biosynthetic route by plants *Artemisia annua* or modified microorganisms is dominating production, a couple of alternative chemical pathways exist, including photochemical 1,2,4-trioxane ring formation from artemisinic acid, a biosynthetically available precursor. The process developed by Sanofi involves the generation of singlet oxygen by tetraphenylporphyrin (TPP) under irradiation by mercury vapor lamps in a semibatch fashion producing up to 370 kg at a time;^76^ however, the production cost of the final product is still too high to compete with natural source-derived substance. One of the lab-scale attempts to improve the reaction used a flow luminescent solar concentrator photomicroreactor and methylene blue as a catalyst under outdoor solar irradiation, producing artemisinin with up to 78% yield.^77^

Atmospheric photocatalytic oxygenation under sunlight has also been employed in the synthesis of small building block molecules. For instance, a series of 5-formyl-1,3-oxadiazoles were prepared by oxidative cyclization of N-propargyl amides under sunlight and an air atmosphere; elemental iodine played a dual role as both sensitizer and intermediate forming a catalyst, in this case, producing valuable aldehyde moieties with only slightly less yields than under monochromatic LED irradiation (80% under sunlight after 16 h, 83% and 86% under 450 and 395 nm LEDs, respectively, in screening conditions).^78^ Another example demonstrated the preparation of 1-oxoesters, multifunctional precursors from readily available 1-bromoesters, using a Ru(II) photocatalyst, with reaction time under sunlight being shortened from 24 h to 9 h to achieve the same yield as with a 24W fluorescent bulb.^79^ Oxidation of heteroatoms is possible as well; a recent publication proved the conversion of protected thiophenols to other valuable sulfur-containing molecules (in the case of solar irradiation, sulfonyl chlorides) by a poly(heptazine imide) carbon nitride catalyst; again, implementation of sunlight allowed shortening the reaction time 4-fold compared to a 50W 465 nm LED.^80^

Sunlight oxidation is not limited to oxygenation reactions and is occasionally employed for aerobic dehydrogenation reactions. One notable example is the use of 2D-COFs as solar photocatalysts in the synthesis of 1,2,4-thiadiazoles by cyclization of corresponding N-guanyl thioureas, N-imidoyl thioureas, and N-imidoyl thioamides, employing atmospheric oxygen as a terminal oxidant.^81^ The role of the oxidant here is to abstract two protons, forming new N–S-bonds. Another reaction of particular interest is the splitting of vicinal diols into corresponding benzaldehydes by carbon nitrides in up to 20 mmol scale runs. This process may serve as a model reaction for photocatalytic lignin transformation.^82^

### 5.2 Redox-Neutral Reactions

**Scheme 4:**
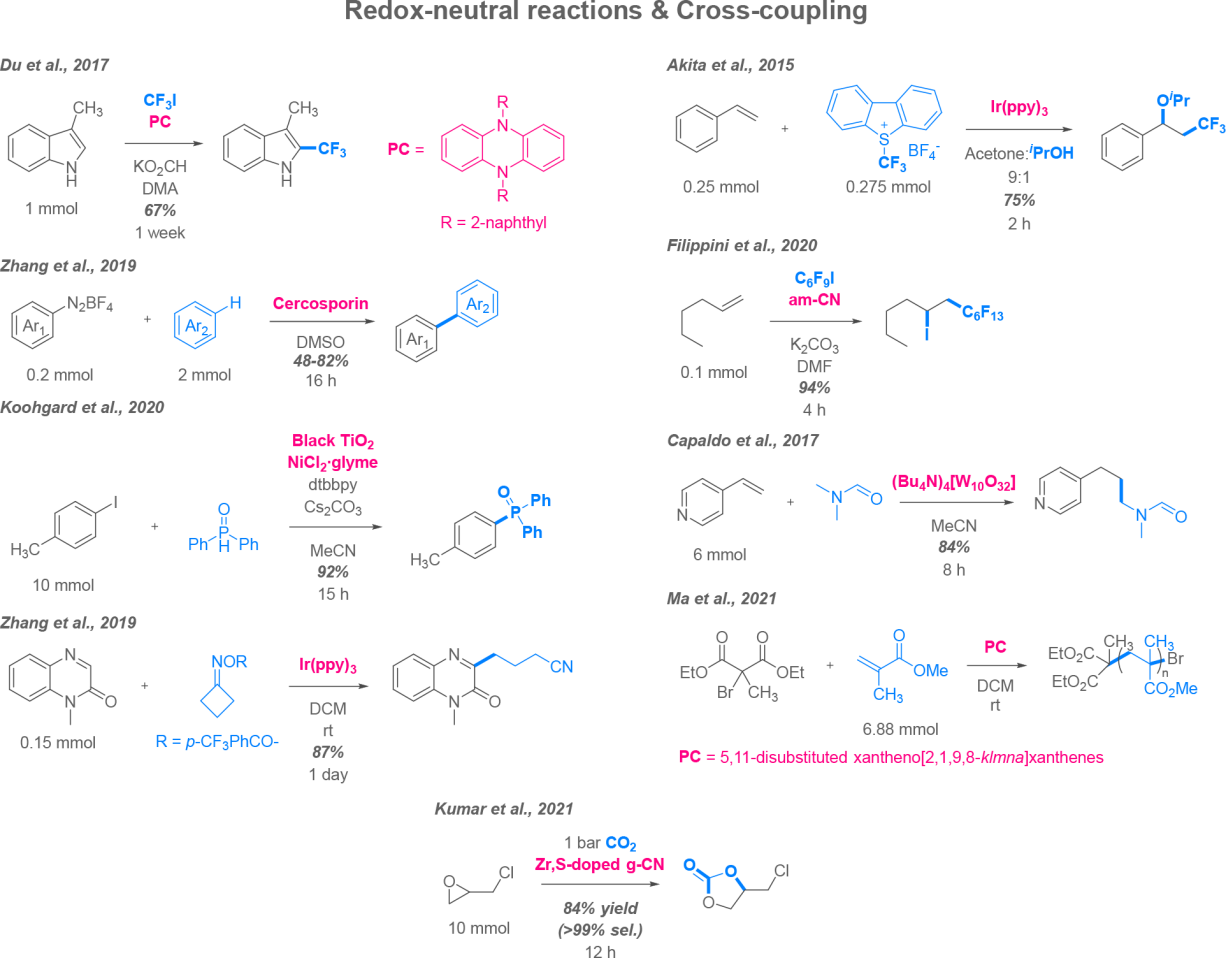
Proof-of-Concept Sunlight-Driven Redox-Neutral Photocatalytic Reactions and Cross-Coupling Used in Organic Synthesis

Redox-neutral reactions here are represented by cross-coupling reactions (including both halide substitution and C–H functionalization reactions), multiple bond additions, and cycle expansions, which allow obtaining complex value-added chemicals with efficiency competitive to more traditional transition metal catalysis protocols (Scheme 4).

Sunlight was employed to drive diverse cross-coupling processes, including trifluoromethylation of indoles with the aid of dihydrophenazine sensitizers,^83^ C–C-coupling of diazonium salts with nonfunctionalized arenes using natural dye as a photocatalyst,^84^ and a dual Ni-photoredox reaction between aryl iodides and disubstituted phosphine oxides.^85^ A distinct feature of these protocols is that they either allow C–H functionalization with only one of the blocks containing leaving groups or replace the palladium(0) catalyst with nickel complexes, making overall transformations more sustainable. Another notable example demonstrated a regioselective introduction of a primary alkyl chain to quinoxalinones by ring opening of cyclic *O*-activated oximes with yields comparable to those achieved with LED light sources.^86^

Regarding ene addition reactions, integration of perfluoroalkanes into aliphatic chains to make functionalized blocks is a study of immediate interest; fluoroalkylated ethers^87^ and alkyl iodides^88^ were successfully obtained using solar photocatalysis. There are also examples of nonfunctionalized carbon addition to vinylpyridines to make N-formyl aminoalkylpyridines, including artificial flavors, using tetrabutylammonium decatungstate. In this case, natural sunlight outperformed a solar simulator (84% yield versus 77%); however, the yield was still higher for a 10 × 15W fluorescent lamp setup (94%).^89^ The most notable reported case of ene reactions is polymerization of a brominated malonic ester “starter” and acryl monomers, where xantheno[2,1,9,8-*klmna*]xanthene photocatalysts are used to maintain chain propagation to give a mass of several kDa for product polymers without utilizing toxic and dangerous radical initiators, although the reaction performed better under blue LED irradiation.^90^

The production of ethylene carbonates from oxiranes and CO_2_ is an important industrial process for making bulk solvents and synthetic blocks.^91^ In a recent publication, carbon dioxide is inserted into an epichlorohydrin cycle photocatalytically using zirconium-thiamine-doped graphitic carbon nitride to produce chloropropylene carbonate with good yield and selectivity and 2 times shorter reaction times than for a 250W Hg lamp.^92^

### 5.3 Outline of Catalytic Systems Used for Photoredox Transformations

From the examples discussed above, some trends of current organic photocatalysis can be outlined. First and foremost, the researchers’ focuses somewhat shift from ruthenium- and iridium-based photocatalysts toward cheaper, more sustainable, and abundant alternatives. More and more photoredox-active compounds find their applications, ranging from well-studied molecules, such as methylene blue and iodine, to natural dyes, synthetic polyaromatic systems, and polytungstate anionic clusters, which can allow for comparable performance without utilization of precious metals.

Another key feature of modern photoredox studies is implementation of heterogeneous semiconductors as recyclable photocatalysts with multiple times usage. One of the most widely known is titanium dioxide, an abundant natural white pigment with a half century history of photocatalytic applications,^93^ that still attracts close attention due to its ability to be easily sensitized or doped with different elements to yield materials with significantly altered band structure and light absorption, which can be used for fine-tuning of its properties.^94−96^ Nonetheless, in the past two decades, other perspective classes of materials appeared, including carbon-based catalysts and 2D nanomaterials.

Carbon surfaces and composites are emerging materials that can be prepared from numerous organic precursors in an environmentally friendly way, providing very high surface areas and good photophysical properties. These materials are yet to find their applications in organic photocatalysis, and current research is mostly focused on different applications such as water splitting^97^ and oxidative waste treatment.^98^

Two-dimensional nanomaterials are a broad class of semiconductors with a layered sheet structure that provides additional possibilities for doping and modification along with enhanced charge separation and migration.^99^ These semiconductors are represented mostly by 2D covalent and metal organic frameworks (2D-COFs, 2D-MOFs) and carbon nitrides, which are established to be versatile materials for carrying out net-oxidative, net-neutral, net-reductive, and dual photoredox transformations.^100−102^

## 6 Overview of Reactors: Examples of Using Reactors to Enable Photocatalytic Reactions

At the present moment, there are less than a dozen gram-scale solar photoreactors that have been applied for multiple reactions, and some of them are still operational. These setups (SOLFIN, SOLARIS/PROPHIS, Sunflow, and more) and targeted transformations have been already covered extensively in several reviews on solar photocatalysis;^67,103,104^ therefore, this section only focuses on more recent reports and emerging technologies.

**Figure 5:**
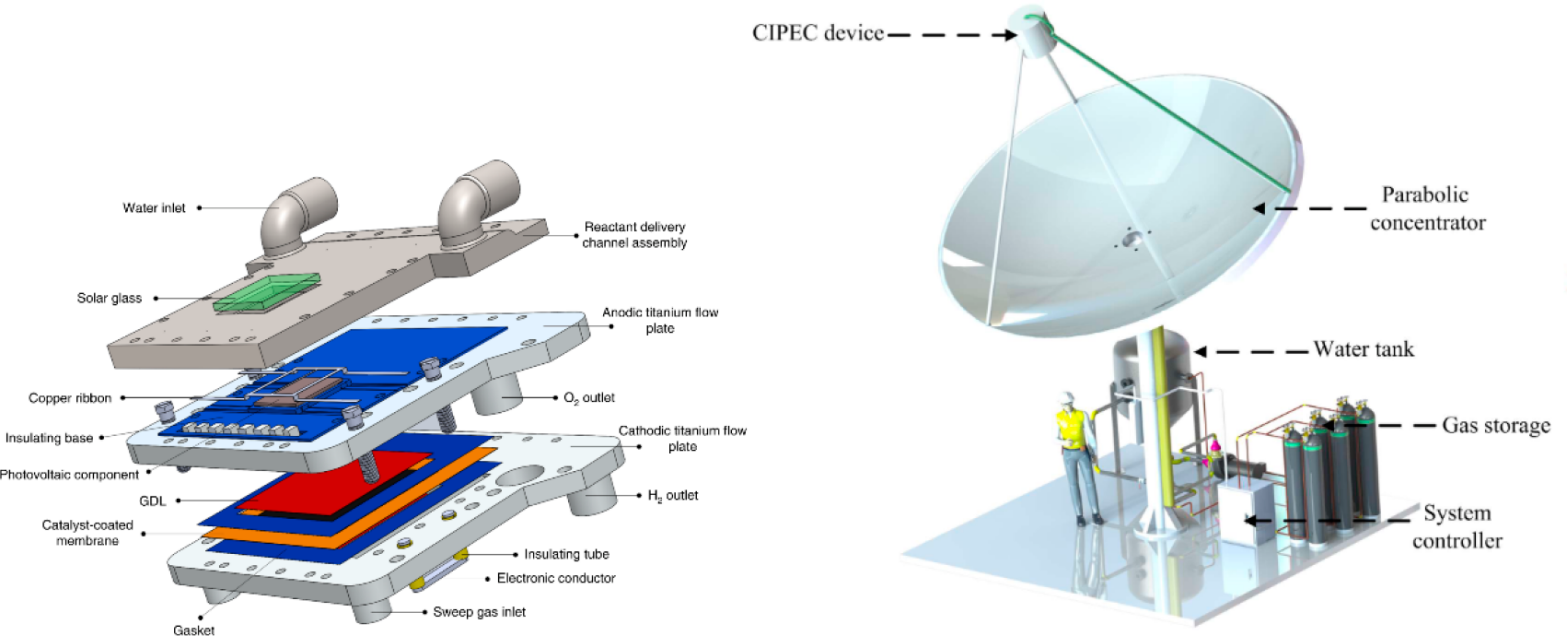
Schematic of integrated PEC unit (left) and illustration of “solar dish” light concentrator device at EPFL (right). Reprinted with permission from ref (105). Copyright 2019, Springer Nature.

Integrated photoelectrochemical (IPEC) devices are commonly utilized in experimental setups for hydrogen evolution, where a photoabsorber is separated from an electrolyte by a thin conductive layer(s) in a flat pack cell. This integrated system of IPEC allows for efficient external cooling and limits energy losses in comparison to divided cells. The compact size of the reactor cells simplifies solar device construction. In EPFL, an IPEC device has been operational since 2019 coupled with a so-called “solar dish”, which mimics radio telescope and satellite dish construction; the mirror concentrates light in a focal spot where IPEC is mounted (Figure 5).^105^ The major benefit of such a design is that the setup could be made more rotationally mobile and be able to track the sun during the whole day, and the reflecting dish provides higher concentration rates per reactor area than linear concentrators. The scale-up of this setup, however, seems a challenging task considering the chip-to-dish surface ratio, the amount of occupied space, and the limited productivity of a compact reactor design. It is also important to note that these particular and similar setups are usually employed for hydrogen evolution and water splitting, so the possibilities of utilizing IPEC devices for organic transformations or CO_2_ reduction are still uncertain and are a subject of further research.

**Figure 6:**
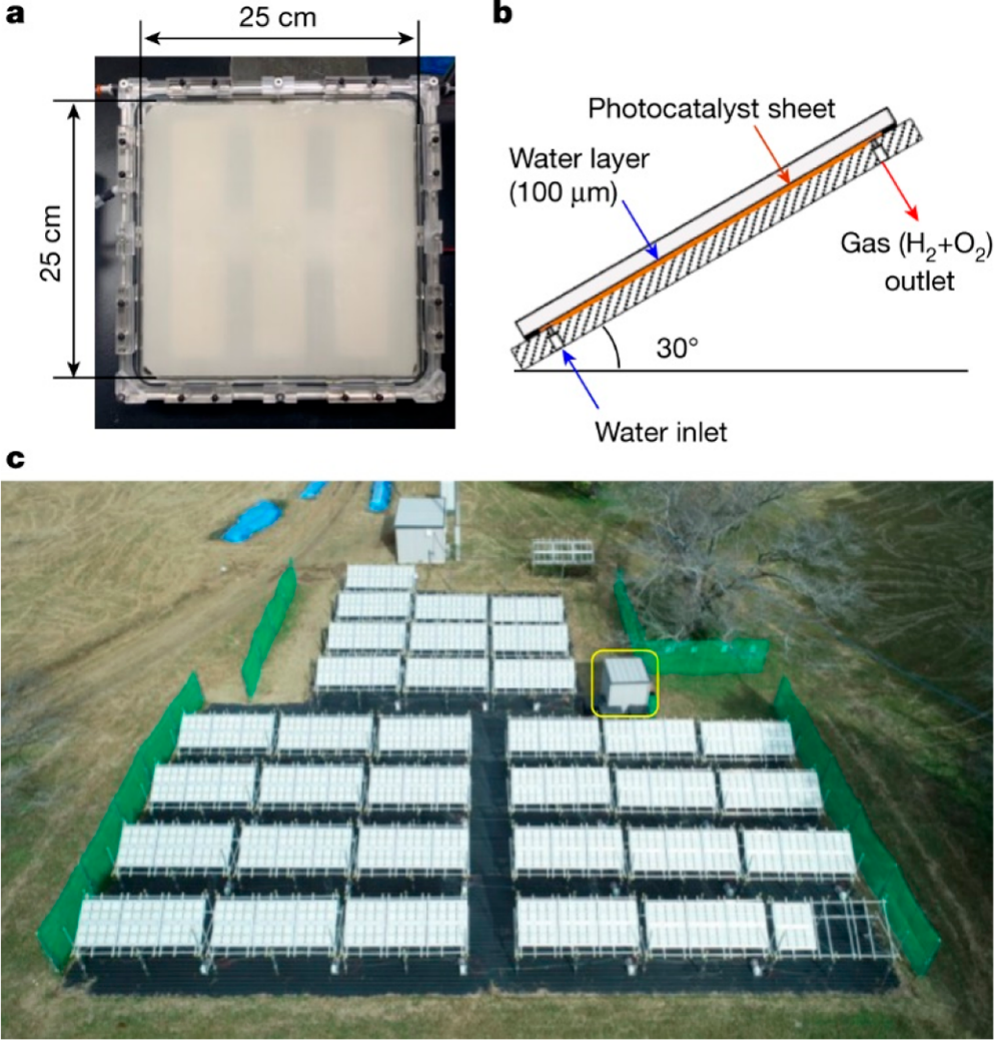
Experimental photocatalytic hydrogen evolution setup at the University of Tokyo. (a) Individual reactor unit (625 cm^2^). (b) Schematic of reactor unit positioning from the side. (c) Overhead view of the entire 100 m^2^ hydrogen production system. Reprinted with permission from ref (106). Copyright 2021, Springer Nature.

Despite the efficiency of solar concentrators in terms of providing higher photon flux per surface unit, their implementation sets certain limitations upon the device construction, making it more complex and harder to maintain in proper condition. To achieve higher productivity with lower cost for a solar chemistry product, concentrators ideally should be avoided by employing a more efficient and stable photocatalyst. A battery of test water-splitting inclined plate collectors (IPCs) was set up recently at the University of Tokyo to support this hypothesis, using the same principles as various water treatment panels (Figure 6).^106^ Each reactor consists of a 625 cm^2^ glass sheet coated with SrTiO_3_:Al particles (<1 μm), which is encapsulated into a UV-transparent casing. The setup is inclined at an angle of 30° from the ground, and water is fed into the bottom part through a 0.1 mm gap between the window and the photocatalytic sheet. During water splitting, it forms a moist hydrogen–oxygen mixture that leaves through an exhaust tube on top of the setup and then is combined with output from other cells and processed in a central unit to remove moisture and oxygen, producing hydrogen with greater than 95% purity. The panel field is arranged from 1600 reactor units, which is 100 m^2^ in total, with peak STH efficiency of 0.76% and output of up to 3.7 L min^–1^ of hydrogen. The productivity of this setup can be further increased by utilizing improved catalysts.

Previously, there were successful attempts to utilize ICP/flatbed reactors for organic synthesis, but the concentrator-based process remains dominating in the field.^107^ A similar reactor is also operational in JCU in Townsville, Australia, using reflecting back surface and homogeneous catalysts.^108^ Still, there is a significant gap between current state-of-the-art flatbed photoreactors for organic syntheses and their industrial implementation as there are issues with poor mass transfer in laminar flow (in general) and in contact between the liquid phase and a catalyst (for reactors with heterogeneous catalyst bed).

### Challenges and Perspectives

Today, the main common obstacle for wide implementation of solar photocatalysis is still a lack of catalysts that can provide commercially acceptable yields and production rates of target compounds. While the hydrogen evolution process is steadily transferred into pilot photocatalytic setups, carbon dioxide reduction is still more of an emerging concept, rather than a ready-to-market technology, which is currently outpaced by a simpler photothermal reduction. To be commercially successful, CO_2_ reduction photocatalysts require very high selectivity toward a certain product, either syngas or methanol, along with structural stability and stable efficiency upon direct air capture technology, and current materials are still to match all these requirements.

Another challenge to be solved in the future is the reactor design; there are competing paradigms for setup organization and scale-up issues. Currently, two main approaches for solar reactor design exist, depending on whether solar concentrators are used or not. This seemingly minor change dictates the properties of the catalyst and some more technical details of the process. Solar photocatalytic panels can uilize a significant amount of irradiation per catalyst mass but require higher efficiency of the latter, while tubular, chamber, and chip devices with solar concentrators are less demanding to catalysts but seem to require more careful phase flow design. Establishing an optimal technological balance between these issues would lead to commercial setups in the fututre.

In the case of complex organic transformations, solar photocatalysis is currently more of a lab-scale, proof-of-concept method; multiple gram-scale runs were reported in the past but never yielded any commercial process. Lack of flexibility and versatility to carry out multiple processes without significant reassembling is the key factor preventing wide implementation of universal reactors for organic synthesis under sunlight, at least at a pilot scale. Regarding actual industrial processes, for example, pharmaceuaticals, sunlight photocatalysis would be the most beneficial for high demand bestselling drugs, as it would allow for significant cost reduction for irradiation. However, this still requires very high efficiency and low capital costs of the photochemical or photoredox process. The notable example is the Sanofi artemisinin process discussed above; the photochemical process was not able to compete with a plant-derived product cost wise, and the whole plant was later sold after a couple years of operation.^109^ One promising solution to resolve this problem would be to eventually shift from ruthenium and irirdium complexes to organic dye photocatalysts and semiconductors for the sake of sustainability and recyclability.

Nonetheless, achieving a stable sunlight economy is far from impossible, as low-cost energy for the chemical industry is an extremely desirable concept with lots and lots of investment provided for this kind of research, which is gradually increasing each year. While it may seem now that current state-of-the-art technology does not satisfy our demands, with this rate of progress, we may reach the first solar plants rather soon, probably by the end of the decade.

## Conclusion

Despite significant efforts in solar fuel research, sunlight photocatalysis can still be considered an emerging industry not mature enough for proper commercialization, and examples of high TRL prototypes and pilot setups are scarce. Recent successes in solar hydrogen evolution and CO_2_ reduction are inspiring, and solar fuel projects tend to receive increased funding. However, demand for catalysts with sufficient efficiency and stability is still not satisfied and thus hinders further progress in these fields, along with uncertainty in the setup design. If these challenges are to be overcome, the future of solar fuels (at least for hydrogen and bulk chemicals) seems quite promising.

## Supporting Information Available

Summary of photochemistry (PC)- and photoelectrochemistry (PEC)-based projects funded by the European Commission since 2009 (PDF)
