# Supplementary material for: Progress in Development of Photocatalytic Processes for Synthesis of Fuels and Organic Compounds under Outdoor Solar Light
Source: Energy Fuels. 2022 Apr 13;36(9):4625–39. doi: 10.1021/acs.energyfuels.2c00178 (PMC9082502; doi:10.1021/acs.energyfuels.2c00178)
Supplement: Supplementary file 1 — ef2c00178_si_001.pdf [file ef2c00178_si_001.pdf]

Progress in Development of Photocatalytic Processes for Synthesis of Fuels and Organic Compounds under Outdoor Solar Light

Alexey Galushchinskiy[a], Roberto González-Gómez[b], Kathryn McCarthy[b], Pau Farràs [b], Aleksandr Savateev[a]**

[a]Department of Colloid Chemistry, Max Planck Institute of Colloids and Interfaces, Am Mühlenberg 1, 14476 Potsdam, Germany

[b]School of Chemistry, Ryan Institute, National University of Ireland, Galway H91 CF50, Ireland

* Email: Oleksandr.Savatieiev@mpikg.mpg.de, pau.farras@nuigalway.ie

Table S1. Photochemistry (PC) and photoelectrochemistry (PEC) based projects funded by the European Commission since 2009

Project	Starting molecule(s)	Target molecule(s)	Start date	Duration	Total Budget	EU contribution	Solar-driven Technology	Funding Call	TRL Scale	Project Website
SOLHYDROMICS (Nanodesigned electrochemical converter of solar energy into hydrogen hosting natural enzymes or their mimics)	Water	Hydrogen	Jan-09	3 years	€3 655 827.74	€2 779 679.00	PEC	FP7-ENERGY-2008-FET	TRL 1-3	Nanodesigned electrochemical converter of solar energy into hydrogen hosting natural enzymes or their mimics | SOLHYDROMICS Project | Results | FP7 | CORDIS | European Commission (europa.eu)
NANOPEC (Nanostructured Photoelectrodes for Energy Conversion)	Water	Hydrogen, Oxygen	Jan-09	2 years	€3 589 188.00	€2 699 909.00	PEC	FP7-ENERGY-NMP-2008-1	TRL 1-3	NanoPEC | EPFL
H2OSPLIT (WATER SPLITTING CATALYSTS FOR ARTIFICIAL PHOTOSYNTHESIS)	Water	Hydrogen, Oxygen	May-09	4 years	€100 000.00	€100 000.00	PC	FP7-PEOPLE-IRG-2008	TRL 1-3	https://cordis.europa.eu/project/id/239199
Hybrid Nanomaterials (Development of Hybrid Nanostructures for Photocatalysis and Fuel Cell Applications)	Water	Hydrogen, Oxygen	Apr-10	4 years	€100 000.00	€100 000.00	PC	FP7-PEOPLE-2009-RG	TRL > 3	https://cordis.europa.eu/project/id/256392

Project	Starting molecule(s)	Target molecule(s)	Start date	Duration	Total Budget	EU contribution	Solar-driven Technology	Funding Call	TRL Scale	Project Website
PHOTOBIO23JC (SYNTHESIS OF NOVEL NANOSTRUCTURED METAL-SUPPORTED PHOTOCATALYSTS: CHARACTERIZATION AND PROMISING APPLICATIONS IN THE PRODUCTION OF HIGH VALUE CHEMICALS FROM LIGNOCELLULOSIC BIOMASS)	Glucose	Glucaric acid, gluconic acid and arabitol	Apr-10	4 years	€100 000.00	€100 000.00	PC	FP7-PEOPLE-2009-RG	TRL 1-3	https://cordis.europa.eu/project/id/256283
SOLAR BIO-HYDROGEN (Design of Hybrid Nanostructured Bio-photocatalyst for Their Application in Bio-photoelectrochemical Hydrogen Production	Carbon Dioxide, Water	Hydrogen, Oxygen, CO	Sep-10	2 years	€181 103.20	€181 103.20	PEC	FP7-PEOPLE-2009-IIF	TRL 1-3	Advanced devices for the Reduction of CArbon DIoxide and Artificial photosynthesis | ARCADIA Project | Fact Sheet | H2020 | CORDIS | European Commission (europa.eu)
PHOTOMEM (Photocatalytic and membrane technology process for olive oil mill waste water treatment)	Waste water	Clean water	Dec-10	2 years	€1 215 075.00	€909 935.75	PC	FP7-SME-2010-1	TRL > 3	https://cordis.europa.eu/project/id/262470
AQUA-PULSE (Photocatalysis with UV LED Sources for Efficient Water Purification)	Waste water	Clean water	Sep-11	2 years	€1 415 933.00	€1 117 218.00	PC	FP7-SME-2011	TRL > 3	https://cordis.europa.eu/project/id/286641
ENERGYSURF (Surfaces of Energy Functional Metal Oxides)	Water	Hydrogen, Oxygen	Oct-11	6 years	€2 364 681.00	€2 364 681.00	PC	ERC-2010-AdG_20100224	TRL 1-3	https://cordis.europa.eu/project/id/267768
PhotoCatMOF (Dye-Sensitized Metal-Organic Frameworks for Photocatalytic Water Splitting)	Water	Hydrogen	Apr-12	2 years	€209 033.40	€209 033.40	PC	FP7-PEOPLE-2011-IEF	TRL 1-3	https://cordis.europa.eu/project/id/299818
ARTIPHYCTION (Fully artificial photo-electrochemical device for low temperature hydrogen production)	Water	Hydrogen	May-12	3 years	€3 594 580.50	€2 187 039.80	PEC	FCH-JU-2011-1	TRL > 3	ARTIPHYCTION

Project	Starting molecule(s)	Target molecule(s)	Start date	Duration	Total Budget	EU contribution	Solar-driven Technology	Funding Call	TRL Scale	Project Website
Solar Fuel by III-Vs (Direct photoelectrochemical generation of solar fuels using dilute nitride III-V compound semiconductor heterostructures on silicon: epitaxy, electrochemistry, and interface characterization)	Water	Hydrogen	Aug-12	3 years	€255 453.00	€255 453.00	PEC	FP7-PEOPLE-2011-IOF	TRL 1-3	Solar Fuel by III-Vs :: European Project :: Up2Europe
PhotoCO2 (Photocatalytic reduction of carbon dioxide into fuels)	Carbon Dioxide	Methanol, Methane, Hydrogen	Sep-12	2 years	€50 000.00	€50 000.00	PEC	FP7-PEOPLE-2011-CIG	TRL 1-3	CORDIS | European Commission (europa.eu)
CO2REDUCTDINUCLEAT (A New Approach to Electrocatalytic CO2 Reduction Based on Supramolecular, Dinucleating Catalysts)	Carbon Dioxide	MeOH, HCHO or OHCOCOOH	Sep-12	2 years	€170 327.10	€170 327.10	PC/EC	FP7-PEOPLE-2011-IOF	TRL 1-3	https://cordis.europa.eu/project/id/299571
CO2PHOTORED (Carbon dioxide photoreduction: A great challenge for photocatalysis)	Carbon Dioxide, Water	Hydrocarbons	Oct-12	Oct-14	€176 053.20	€176 053.20	PC	FP7-PEOPLE-2011-IEF	TRL > 3	https://cordis.europa.eu/project/id/298740
carbenergy (Mesoionic carbene complexes for water splitting: Harnessing renewable energy sources)	Water	Hydrogen	Oct-12	1 years	€150 786.00	€136 075.85	PEC	ERC-2012-PoC	TRL > 3	https://cordis.europa.eu/project/id/324609
HETMAT (Heterostructure Nanomaterials for Water Splitting)	Water	Hydrogen	Nov-12	4 years	€100 000.00	€100 000.00	PC	FP7-PEOPLE-2012-CIG	TRL > 3	https://cordis.europa.eu/project/id/322114
PHOCSCLEEN (PHOtoCathalytic Systems for CLean Energy and Environment Applications)	Water	Hydrogen	Nov-12	4 years	€163 800.00	€163 800.00	PC	FP7-PEOPLE-2012-IRSES	TRL > 3	https://cordis.europa.eu/project/id/318977
PHOCS (Photogenerated Hydrogen by Organic Catalytic Systems)	Water	Hydrogen	Dec-12	3 years	€3 828 934.90	€2 849 000.00	PEC	FP7-ENERGY-2012-1-2STAGE	TRL > 3	www.phocs.eu
ECO2CO2 (Eco-friendly biorefinery fine chemicals from CO2 photo-catalytic reduction)	Carbon Dioxide	Fine chemicals (fragrances, flavourings, adhesives, monomers,…)	Dec-12	4 years	€4 711 872.27	€3 424 438.00	PEC	FP7-NMP-2012-SMALL-6	TRL > 3	https://cordis.europa.eu/project/id/309701

Project	Starting molecule(s)	Target molecule(s)	Start date	Duration	Total Budget	EU contribution	Solar-driven Technology	Funding Call	TRL Scale	Project Website
photocatH2ode (Gathering organic and hybrid photovoltaics with artificial photosynthesis for Photo-Electro-Chemical production of hydrogen)	Water	Hydrogen	Dec-12	5 years	€1 500 000.00	€1 500 000.00	PEC	ERC-2012-StG_20111012	TRL > 3	Gathering organic and hybrid photovoltaics with artificial photosynthesis for Photo-Electro-Chemical production of hydrogen | photocatH2ode Project | Fact Sheet | FP7 | CORDIS | European Commission (europa.eu)
CYCLICCO2R (Production of Cyclic Carbonates from CO2 using Renewable Feedstocks)	Carbon Dioxide, diols	Cyclic carbonates	Jan-13	3 years	€5 254 690.80	€3 851 934.00	PC/EC	FP7-NMP-2012-SMALL-6	TRL > 3	http://www.cyclicco2r.eu/.
SOLAROGENIX (Visible-Light Active Metal Oxide Nano-catalysts for Sustainable Solar Hydrogen Production)	Water	Hydrogen	Feb-13	3 years	€3 906 486.00	€2 755 708.00	PEC	FP7-NMP-2012-SMALL-6	TRL > 3	http://www.solarogenix.eu
PCATDES (Photocatalytic Materials for the Destruction of Recalcitrant Organic Industrial Waste)	Waste water	Clean water	Feb-13	4 years	€5 148 336.61	€3 954 395.00	PC	FP7-NMP-2012-SMALL-6	TRL > 3	https://cordis.europa.eu/project/id/309846
RPSII (Re-wiring of photosystem II enzymes to metal-oxide electrodes in artificial photosynthetic devices for enhanced photocatalytic water splitting performance)	Water	Hydrogen	Mar-13	2 years	€221 606.40	€221 606.40	PC	FP7-PEOPLE-2012-IIF	TRL 1-3	https://cordis.europa.eu/project/id/328085
COCHALPEC (Development of electrodes based on copper chalcogenide nanocrystals for photoelectrochemical energy conversion)	Water	Hydrogen	Jun-13	2 years	€184 709.40	€184 709.40	PEC	FP7-PEOPLE-2012-IEF	TRL 1-3	https://cordis.europa.eu/project/id/326919
PHOTOSURF (Investigating the 2D Self-Assembly of Photo-sensitive Molecules on Semiconductor and Insulating Surfaces)		Solar fuels	Sep-13	4 years	€100 000.00	€100 000.00	PC	FP7-PEOPLE-2013-CIG	TRL 1-3	https://cordis.europa.eu/project/id/618777
SusNano (Sustainable Nanocomposites for Photocatalysis)	Carbon Dioxide, Water	Hydrogen/hydrocarbons	Oct-13	5 years	€100 000.00	€100 000.00	PC	FP7-PEOPLE-2012-CIG	TRL 1-3	https://cordis.europa.eu/project/id/334302

Project	Starting molecule(s)	Target molecule(s)	Start date	Duration	Total Budget	EU contribution	Solar-driven Technology	Funding Call	TRL Scale	Project Website
GRAPES (GRaphene Enhancement of the Photocatalytic Activity of Semiconductors)	Water	Clean water/air, solar fuels (H2)	Dec-13	2 years	€221 606.40	€221 606.40	PC	FP7-PEOPLE-2012-IEF	TRL 1-3	https://cordis.europa.eu/project/id/329945
TripleSolar (Solar Energy Conversion in Molecular Multi-Junctions)	Carbon Dioxide, Water	Hydrogen, Oxygen	Mar-14	5 years	€2 493 585.00	€2 493 585.00	PEC	FP7-IDEAS-ERC	TRL > 3	Solar Energy Conversion in Molecular Multi-Junctions | TripleSolar Project | Fact Sheet | FP7 | CORDIS | European Commission (europa.eu)
CO2SF (Solar Fuel Chemistry: Design and Development of Novel Earth-abundant Metal complexes for the Photocatalytic Reduction of Carbon Dioxide)	Carbon Dioxide, Water	Hydrogen and Carbon Monoxide	Mar-14	2 years	€299 558.40	€299 558.40	PC/PEC	FP7-PEOPLE-2013-IIF	TRL 1-3	Novel materials for solar fuel technology | CO2SF Project | Results in brief | FP7 | CORDIS | European Commission (europa.eu)
FOPS-water (Fundamentals Of Photocatalytic Splitting of Water)	Water	Hydrogen	Mar-14	5 years	€1 498 800.00	€1 498 800.00	PC	ERC-2013-StG	TRL 1-3	https://cordis.europa.eu/project/id/336679
PECDEMO (Photoelectrochemical Demonstrator Device for Solar Hydrogen Generation)	Water	Hydrogen	Apr-14	3 years	€3 337 682.79	€1 830 644.00	PEC	FCH-JU-2013-1	TRL > 3	www.pecdemo.eu
ETASECS (Extremely Thin Absorbers for Solar Energy Conversion and Storage)	Water	Hydrogen, Oxygen	Sep-14	5 years	€2 150 000.00	€2 150 000.00	PEC	ERC-2013-CoG	TRL > 3	https://cordis.europa.eu/project/id/617516
CO2TOSYNGAS (Visible-light-driven CO2 reduction to SynGas using water as electron and proton donor over a Z-scheme photoelectrochemical cell)	Carbon Dioxide	CO/ Hydrogen, hydrocarbons	Oct-14	3 years	€231 283.20	€231 283.20	PEC	FP7-PEOPLE-2013-IEF	TRL 1-3	https://cordis.europa.eu/project/id/623061
SolHyPro (Water splitting by solar energy: From lab-scale to prototype devices)	Water	Hydrogen	Jun-15	2 years	€170 509.20	€170 509.20	PEC	H2020-MSCA-IF-2014	TRL > 3	https://cordis.europa.eu/project/id/656132
DIACAT (Diamond materials for the photocatalytic conversion of CO2 to fine chemicals and fuels using visible light)	Carbon Dioxide	Organic chemicals	Jul-15	4 years	€3 872 981.25	€3 872 981.25	PC	H2020-FETOPEN-2014-2015-RIA	TRL 1-3	Diamond materials for the photocatalytic conversion of CO2 to fine chemicals and fuels using visible light | DIACAT Project | Fact Sheet | H2020 | CORDIS | European Commission (europa.eu)

Project	Starting molecule(s)	Target molecule(s)	Start date	Duration	Total Budget	EU contribution	Solar-driven Technology	Funding Call	TRL Scale	Project Website
HyMAP (Hybrid Materials for Artificial Photosynthesis)	Carbon Dioxide, Water	Methanol, Methane, Hydrogen	Jul-15	7 years	€2 506 738.00	€2 506 738.00	PC	ERC-2014-CoG	TRL 1-3	Home - HyMap
GREENLIGHT_REDCAT (Towards a Greener Reduction Chemistry by Using Cobalt Coordination Complexes as Catalysts and Light-driven Water Reduction as a Source of Reductive Equivalents)	Water, Carbon Dioxide, Ketones, Aldehydes, Olefins	CO, Alcohols	Jul-15	5 years	€1 999 063.00	€1 999 063.00	PC	ERC-2014-CoG	TRL 1-3	Towards a Greener Reduction Chemistry by Using Cobalt Coordination Complexes as Catalysts and Light-driven Water Reduction as a Source of Reductive Equivalents | GREENLIGHT_REDCAT Project | Fact Sheet | H2020 | CORDIS | European Commission (europa.eu)
BioAqua (Water as cosubstrate for biocatalytic redox reactions)	Water and Visible Light	Oxidoreductases-catalysed transformations	Jul-15	5 years	€1 998 020.00	€1 998 020.00	PC	ERC-2014-CoG	TRL 1-3	https://cordis.europa.eu/project/id/648026
COFLeaf (Fuel from sunlight: Covalent organic frameworks as integrated platforms for photocatalytic water splitting and CO2 reduction)	Carbon Dioxide, Water	Hydrogen	Sep-15	5 years	€1 497 125.00	€1 497 125.00	PC	ERC-2014-STG	TRL 1-3	https://cordis.europa.eu/project/id/639233
Supramol (Towards Artificial Enzymes: Bio-inspired Oxidations in Photoactive Metal-Organic Frameworks)	Alkenes, Alcohols, water	Hydrogen	Sep-15	6 years	€1 979 366.00	€1 979 266.00	PC	ERC-2014-CoG	TRL 1-3	https://cordis.europa.eu/project/id/647719
Heteroepitaxial α-Fe2O3 photoanodes for solar water splitting	Water	Hydrogen	Oct-15	2 years	€170 509.20	€170 509.20	PEC	H2020-MSCA-IF-2014	TRL 1-3	EU H2020 Project "EPIANODES (Heteroepitaxial α-Fe2O3 photoanodes for solar water splitting)": description, partecipants, costs and EC-fundings (fabiodisconzi.com)
SOLENALGAE (IMPROVING PHOTOSYNTHETIC SOLAR ENERGY CONVERSION IN MICROALGAL CULTURES FOR THE PRODUCTION OF BIOFUELS AND HIGH VALUE PRODUCTS)	Biomass	biofuels	Mar-16	5 years	€1 441 875.00	€1 441 875.00	PC	ERC-2015-STG	TRL > 3	https://cordis.europa.eu/project/id/679814
ARCADIA (Advanced devices for the Reduction of CArbon DIoxide and Artificial photosynthesis)	Carbon Dioxide, Water	Hydrogen and CO	May-16	2 years	€168 277.20	€168 277.20	PEC	H2020-MSCA-IF-2015	TRL 1-3	https://cordis.europa.eu/project/id/705723

Project	Starting molecule(s)	Target molecule(s)	Start date	Duration	Total Budget	EU contribution	Solar-driven Technology	Funding Call	TRL Scale	Project Website
FANOEC (Fundamentals and Applications of Inorganic Oxygen Evolution Catalysts)	Water	Hydrogen	Jul-16	5 years	€2 199 983.00	€2 199 983.00	PEC	ERC-2015-CoG	TRL 1-3	https://cordis.europa.eu/project/id/681292
ZESMO (ZEolitic reactor hosting Subphthalocyanines and Metal Oxides as photocatalytic system for opto-electronic applications)	Carbon Dioxide, Water	Methane	Sep-16	2 years	€158 121.60	€158 121.60	PC	H2020-MSCA-IF-2015	TRL > 3	https://cordis.europa.eu/project/id/709023
NanoINCAGE (Luminescent Nanocrystals in a Cage for Solar-to-Fuel Conversion)	Carbon Dioxide	ethylene and methane	Sep-16	2 years	€175 419.60	€175 419.60	PEC	H2020-MSCA-IF-2015	TRL 1-3	https://cordis.europa.eu/project/id/701745
PHOTOTRAIN (ENTREPRENEURING DYNAMIC SELF-ORGANIZED INTERFACES IN PHOTOCATALYSIS: A MULTIDISCIPLINARY TRAINING NETWORK CONVERTING LIGHT INTO PRODUCTS)	Carbon Dioxide, Water	Stereoselective organocatalytic transformations/ solar fuels	Oct-16	4 years	€3 630 212.28	€3 630 212.28	PC	H2020-MSCA-ITN-2016	TRL > 3	https://cordis.europa.eu/project/id/722591
MatEnSAP (Semi-Artificial Photosynthesis with Wired Enzymes)	Carbon Dioxide, Water	Formate	Oct-16	7 years	€1 960 289.00	€1 960 289.00	PEC	ERC-2015-CoG	TRL 1-3	https://cordis.europa.eu/project/id/682833/reporting
CATA-LUX (Light-Driven Asymmetric Organocatalysis)	Toluene	Chiral Molecules	Nov-16	5 years	€2 000 000.00	€2 000 000.00	PC	ERC-2015-CoG	TRL 1-3	https://cordis.europa.eu/project/id/681840
A-LEAF (An Artificial Leaf: a photo-electro-catalytic cell from earth-abundant materials for sustainable solar production of CO2-based chemicals and fuels)	Carbon Dioxide, Water	Formate and Oxygen	Jan-17	4 years	€7 980 861.25	€7 980 861.25	PEC	FETPROACT-2016	TRL 1-3	http://www.a-leaf.eu/
PECSYS (Technology demonstration of large-scale photo-electrochemical system for solar hydrogen production)	Water	Hydrogen	Jan-17	3 years	€24 999 992.50	€24 999 992.50	PEC	H2020-JTI-FCH-2016-1	TRL > 3	https://cordis.europa.eu/project/id/735218

Project	Starting molecule(s)	Target molecule(s)	Start date	Duration	Total Budget	EU contribution	Solar-driven Technology	Funding Call	TRL Scale	Project Website
HybridSolarFuels (Efficient Photoelectrochemical Transformation of CO2 to Useful Fuels on Nanostructured Hybrid Electrodes)	Carbon Dioxide	Methanol, CO, Ethanol, formic acid	Jan-17	5 years	€1 498 750.00	€1 498 750.00	PEC	ERC-2016-STG	TRL 1-3	https://cordis.europa.eu/article/id/430093-hybrid-photoelectrodes-advance-solar-fuel-generation
p-TYPE (Transparent p-type semiconductors for efficient solar energy capture, conversion and storage)	Carbon Dioxide, Water	Hydrogen	Jan-17	5 years	€1 499 840.00	€1 499 840.00	PEC	ERC-2016-STG	TRL 1-3	https://cordis.europa.eu/project/id/715354
MOFcat (Fundamental and Applied Science on Molecular Redox-Catalysts of Energy Relevance in Metal-Organic Frameworks)	Carbon Dioxide, Water	Hydrogen	Jan-17	4 years	€1 968 750.00	€1 968 750.00	PEC	ERC-2015-CoG	TRL 1-3	Fundamental and Applied Science on Molecular Redox-Catalysts of Energy Relevance in Metal-Organic Frameworks | MOFcat Project | Fact Sheet | H2020 | CORDIS | European Commission (europa.eu)
IRS-PEC (Elucidating the water photo-oxidation mechanism by infrared spectroscopy)	Water	Oxygen	Feb-17	2 years	€165 598.80	€165 598.80	PEC	H2020-MSCA-IF-2015	TRL 1-3	https://cordis.europa.eu/project/id/708874
2D-COF-WS (Designing and screening two dimensional covalent organic frameworks for effective water splitting)	Water	Hydrogen	Apr-17	2 years	€171 460.80	€171 460.80	PC	H2020-MSCA-IF-2016	TRL 1-3	https://cordis.europa.eu/project/id/751848
SOFT-PHOTOCONVERSION (Solar Energy Conversion without Solid State Architectures: Pushing the Boundaries of Photoconversion Efficiencies at Self-healing Photosensitiser Functionalised Soft Interfaces)	Water	Hydrogen, Oxygen	Apr-17	5 years	€1 499 043.75	€1 499 043.75	PEC	ERC-2016-STG	TRL 1-3	https://cordis.europa.eu/project/id/716792
CO2 Intermediates (From CO2, Water and Sunlight to Valuable Solar Fuels: Tracking Reaction Intermediates in Solar Fuel Generation with Ultrafast Spectroscopy for More Efficient Catalysis)	Carbon Dioxide, Water	CO2 intermediates	May-17	2 years	€183 454.80	€183 454.80	PEC	H2020-MSCA-IF-2016	TRL 1-3	EU H2020 Project "CO2 INTERMEDIATES (From CO2, Water and Sunlight to Valuable Solar Fuels: Tracking Reaction..)": description, partecipants, costs and EC-fundings (fabiodisconzi.com)

Project	Starting molecule(s)	Target molecule(s)	Start date	Duration	Total Budget	EU contribution	Solar-driven Technology	Funding Call	TRL Scale	Project Website
SCHiMAT (Silicon Cluster based Hierarchical photocatalysts produced by MATrix assembly cluster source)			Jun-17	2 years	€183 454.80	€183 454.80	PC	H2020-MSCA-IF-2016	TRL 1-3	https://cordis.europa.eu/project/id/752102
watersplit (Producing hydrogen by water splitting)	Water	Hydrogen	Jul-17	1 years	€150 000.00	€150 000.00	PEC	ERC-2017-PoC	TRL 1-3	https://cordis.europa.eu/project/id/764203
WHIPCAT (van der Waals Heterostructures for Innovative PhotoCATalysts)	Water	Hydrogen, Oxygen	Sep-17	2 years	€183 454.80	€183 454.80	PEC	H2020-MSCA-IF-2016	TRL 1-3	van der Waals Heterostructures for Innovative PhotoCATalysts | WHIPCAT Project | Fact Sheet | H2020 | CORDIS | European Commission (europa.eu)
PHAROS (Photocatalytic Generation of CarbAnions for Organic Synthesis)	Neutral molecules	Carbanions	Sep-17	5 years	€2 458 200.00	€2 458 200.00	PC	ERC-2016-ADG	TRL 1-3	https://cordis.europa.eu/project/id/741623
AQUAlity (Interdisciplinar cross-sectoral approach to effectively address the removal of contaminants of emerging concern from water)	Contaminants of emerging concern (CECs)	Clean water	Oct-17	5 years	€3 897 678.24	€3 897 678.24	PC	H2020-MSCA-ITN-2017	TRL > 3	https://cordis.europa.eu/project/id/765860
CLAIM (Cleaning Litter by developing and Applying Innovative Methods in european seas)	Microplastics	Clean water	Nov-17	5 years	€6 150 475.25	€5 652 911.01	PC	H2020-BG-2017-1	TRL > 3	https://cordis.europa.eu/project/id/774586
CO2RED (Sunlight driven carbon-dioxide reduction: Hybrid catalytic systems consisting of molecular catalysts and light-harvesting Quantum-dots and semiconductors)	Carbon Dioxide, Water	CO and Hydrogen	Mar-18	2 years	€195 454.80	€195 454.80	PC/ PEC	H2020-MSCA-IF-2016	TRL 1-3	https://cordis.europa.eu/project/id/745604/reporting
PHOTOCHIRO (Photocatalysis to crop protection: asymmetric cascades for chiral heterocycles)	Organic acids, alkenes and enals	Enantioenriched chiral piperidines	Mar-18	2 years	€170 121.60	€170 121.60	PC	H2020-MSCA-IF-2017	TRL > 3	https://cordis.europa.eu/project/id/794211
eSCALED (European School on Artificial Leaf : Electrodes Devices)	Carbon Dioxide, Water	Hydrogen, Oxygen	Apr-18	4 years	€3 599 022.31	€3 599 022.31	PEC	H2020-MSCA-ITN-2017	TRL > 3	https://cordis.europa.eu/project/id/765376

Project	Starting molecule(s)	Target molecule(s)	Start date	Duration	Total Budget	EU contribution	Solar-driven Technology	Funding Call	TRL Scale	Project Website
PHOTO ORGANO-Ir CAT (Photochemical cascade reactions by merging organo- and iridium catalysis: A stereocontrolled entry to molecular complexity.)		Chiral molecules of biological interest	Apr-18	2 years	€158 121.60	€158 121.60		H2020-MSCA-IF-2017	TRL 1-3	https://cordis.europa.eu/project/id/795793
MOSPHotocat (Application of Metal Oxide Semiconductors in Photocatalysis)	Visible light	C-C and C-N linkages in organic molecules	Jul-18	2 years	€177 598.80	€177 598.80	PC	H2020-MSCA-IF-2017	TRL > 3	https://cordis.europa.eu/project/id/793677
PREMHYDRO (PROBING REACTION MECHANISMS IN PHOTOCATALYTIC H2 GENERATION)	Carbon Dioxide, Water	Hydrogen	Sep-18	2 years	€175 866.00	€175 866.00	PC	H2020-MSCA-IF-2017	TRL > 3	PROBING REACTION MECHANISMS IN PHOTOCATALYTIC H2 GENERATION | PREMHYDRO Project | Fact Sheet | H2020 | CORDIS | European Commission (europa.eu)
CO2SPLITTING (Carbon dioxide splitting into higher-value chemicals with hybrid photocatalyst sheets)	Carbon Dioxide	Syngas/alcohols	Sep-18	2 years	€183 454.80	€183 454.80	PC	H2020-MSCA-IF-2017	TRL > 3	Carbon dioxide splitting into higher-value chemicals with hybrid photocatalyst sheets | CO2SPLITTING Project | Fact Sheet | H2020 | CORDIS | European Commission (europa.eu)
ORTHOCAT (Bioorthogonal Photocatalytic Activation of Metal-Based Prodrugs)	Riboflavin	Pt(IV) anticancer prodrugs inside the mitochondria of tumour cells	Sep-18	2 years	€158 121.60	€158 121.60	PC	H2020-MSCA-IF-2017	TRL 1-3	https://cordis.europa.eu/project/id/793702
LIONCAT (LIGHT-PROMOTED, IRON-CATALYSED FORMAL HYDROGENATION OF ORGANIC COMPOUNDS)	Alkene/Alkynes	Hydrocarbons	Oct-18	2 years	€183 454.80	€183 454.80	PC	H2020-MSCA-IF-2017	TRL 1-3	https://cordis.europa.eu/project/id/799664
TECHNOTRAIN (Enabling TECHNOlogies-driven chemistry: a tailored TRAINing research program for batch and flow synthesis of chiral amino derivatives)	Imines, nitroesters	Enantiomerically pure, functionalized amino derivatives	Nov-18	4 years	€784 499.04	€784 499.04	PC	H2020-MSCA-ITN-2018	TRL > 3	https://cordis.europa.eu/project/id/812944
SoFiA (Soap Film based Artificial Photosynthesis)	Carbon Dioxide	Solar fuels	Jan-19	4 years	€3 235 280.00	3205280	PC	H2020-FETOPEN-2018-2019-2020-01	TRL > 3	https://cordis.europa.eu/project/id/828838
Bac-To-Fuel (BACterial conversion of CO2 and renewable H2 inTO bioFUELs)	Carbon Dioxide, Water	Ethanol/Butanol	Jan-19	3 years	€2 999 922.50	€2 999 922.50	PC	H2020-LC-SC3-2018-Joint-Actions-3	TRL > 3	Home | BAC-TO-FUEL (bactofuel.eu)

Project	Starting molecule(s)	Target molecule(s)	Start date	Duration	Total Budget	EU contribution	Solar-driven Technology	Funding Call	TRL Scale	Project Website
Sun2Hy (Sun to Hydrogen)	Water	Hydrogen	Jan-19	5 years?	€1 929 906.00	€1 929 906.00	PEC		TRL > 3	https://renewablesnow.com/news/repsol-enagas-secure-eu-funds-for-photoelectrocatalytic-hydrogen-production-749720/
PECREGEN (Photoelectrochemical Hydrogen Production from H2S in a Regenerative Scrubber)	Hydrogen Sulfide	Hydrogen	Jan-19	1.5 years	€150 000.00	€150 000.00	PEC	ERC-2018-PoC	TRL > 3	https://cordis.europa.eu/project/id/825117
N-STRAINED (Nitrogen-Radical-Based Radical Strain-Release Strategies for the Divergent Assembly of Polyfunctionalized 3D-Building Blocks)	strained hydrocarbons (eg propellane)	polyfunctionalized bicyclo[1.1.1]pentylamines	Apr-19	2 years	€212 933.76	€212 933.76	PC	H2020-MSCA-IF-2018	TRL 1-3	https://cordis.europa.eu/project/id/842422
H2O-SPLIT (Carbon-Oxynitride Coupled Artificial Photosynthesis System For Solar Water Splitting Beyond 600 nm)	Water	Hydrogen	May-19	2 years	€171 460.80	€171 460.80	PC	H2020-MSCA-IF-2017	TRL > 3	https://cordis.europa.eu/project/id/793882
AtropFluoPhoto (Stereoselective Synthesis of Atropisomeric Fluorophores for Asymmetric Photocatalysis)		Chiral Heterocyclic Fluorophores	May-19	2 years	€203 149.44	€203 149.44	PC	H2020-MSCA-IF-2018	TRL 1-3	https://cordis.europa.eu/project/id/840456
PhotoCatRed (Visible-light-driven Photocatalytic CO2 Reduction to Solar fuels by multinary N-Graphene based Heterostructure Composites)	Carbon Dioxide	Solar fuels	Aug-19	2 years	€162 040.32	€162 040.32	PC	H2020-MSCA-IF-2018	TRL 1-3	EU H2020 Project "PHOTOCATRED (Visible-light-driven Photocatalytic CO2 Reduction to Solar fuels by multinary..)": description, partecipants, costs and EC-fundings (fabiodisconzi.com)
QuantumSolarFuels (Photoelectrochemical Solar Light Conversion into Fuels on Colloidal Quantum Dots Based Photoanodes)	Water	Hydrogen	Nov-19	3 years	€237 768.00	€237 768.00	PEC	H2020-MSCA-IF-2018	TRL > 3	https://cordis.europa.eu/project/id/846107

Project	Starting molecule(s)	Target molecule(s)	Start date	Duration	Total Budget	EU contribution	Solar-driven Technology	Funding Call	TRL Scale	Project Website
SOLAR2CHEM (Training the next generation of scientists in solar chemicals for a sustainable Europe by hybrid molecule/semiconductor devices)	Visible light	Solar chemicals	Feb-20	4 years	€4 037 074.04	€4 037 074.04	PC	H2020-MSCA-ITN-2019	TRL > 3	https://cordis.europa.eu/project/id/861151
PEC_Flow (Continuous-flow Photoelectrochemical Cells for Carbon Dioxide Valorization)	Carbon Dioxide, Water	Base chemicals	Feb-20	1 years	€150 000.00	€150 000.00	PEC	ERC-2019-PoC	TRL > 3	https://cordis.europa.eu/project/id/899747
C[Au]PSULE (Crystal phase engineering of Au nanoparticles for enhanced solar fuel generation)	Carbon Dioxide, Water	Fuel	Apr-20	2 years	€166 320.00	€166 320.00	PC	H2020-MSCA-IF-2019	TRL 1-3	Crystal phase engineering of Au nanoparticles for enhanced solar fuel generation | C[Au]PSULE Project | Fact Sheet | H2020 | CORDIS | European Commission (europa.eu)
DECADE (DistributEd Chemicals And fuels production from CO2 in photoelectrocatalytic DEvices)	Carbon Dioxide, alcohols	Ethyl Acetate, ethyl formate	May-20	4 years	€5 358 672.49	€5 198 756.69	PEC	H2020-NMBP-ST-IND-2019	TRL > 3	DistributEd Chemicals And fuels production from CO2 in photoelectrocatalytic DEvices | DECADE Project | Fact Sheet | H2020 | CORDIS | European Commission (europa.eu)
SunCoChem (Photoelectrocatalytic device for SUN-driven CO2 conversion into green CHEMicals)	Carbon Dioxide	Glycolic acid, valeraldehyde, LimoxalTM	May-20	4 years	€6 771 145.00	€6 617 645.00	PC	H2020-NMBP-ST-IND-2019	TRL > 3	https://cordis.europa.eu/project/id/862192
FLOWPHOTOCHEM (Heterogenous Photo(electro)catalysis in Flow using Concentrated Light: modular integrated designs for the production of useful chemicals)	Carbon Dioxide	Ethylene, other high-value chemicals	Jun-20	4 years	€6 993 315.00	€6 993 315.00	PEC	H2020-NMBP-ST-IND-2019	TRL > 3	https://www.flowphotochem.eu/
NATIOMEM (Nano-structured TiON Photo-Catalytic Membranes for Water Treatment)	Waste water	Clean water	Jul-20	3 years	€4 093 309.50	€2 993 230.00	PC	FP7-NMP-2009-SMALL-3	TRL > 3	https://cordis.europa.eu/project/id/245513
SECANS (Solar-to-Chemical Energy Conversion with Advanced Nitride Semiconductors)	Carbon Dioxide	Chemical fuels	Jul-20	5 years	€1 933 750.00	€1 933 750.00	PC	ERC-2019-COG	TRL 1-3	Solar-to-Chemical Energy Conversion with Advanced Nitride Semiconductors | SECANS Project | Fact Sheet | H2020 | CORDIS | European Commission (europa.eu)

Project	Starting molecule(s)	Target molecule(s)	Start date	Duration	Total Budget	EU contribution	Solar-driven Technology	Funding Call	TRL Scale	Project Website
LICROX (Light assisted solar fuel production by artificial CO2 Reduction and water Oxidation)	Carbon Dioxide, Water	Solar fuel	Sep-20	3 years	€3 199 602.50	€3 199 602.50	PEC	H2020-EIC-FETPROACT-2019	TRL > 3	https://cordis.europa.eu/project/id/951843
LIFE VISIONS (InnoVative photocatalytIc paintS for healthy envirOnment and eNergy Saving)	Air pollutants	Clean air	Sep-20	3 years	€1 403 752.00	€757 763.00	PC		TRL > 3	LIFE VISIONS – Life Visions Official Website
SUN2CHEM (Developing solutions for efficient, solar-driven CO2 reduction)	Carbon Dioxide	Ethylene	Oct-20	3 years	€3 941 507.50	€3 941 507.50	PEC/ PC	H2020-LC-SC3-2019-NZE-RES-CC	TRL > 3	https://cordis.europa.eu/project/id/884444
SolarFUEL (Gas Diffusion Electrodes and Flow Cells for Photoelectrochemical CO2 Conversion into Multicarbon Alcohols)	Carbon Dioxide, Water	Multi- carbon Alcohols	Dec-20	2 years	€212 933.76	€212 933.76	PEC	H2020-MSCA-IF-2018	TRL 1-3	https://cordis.europa.eu/project/id/839763
STEPforGGR (Solar up-draft tower to enable atmospheric photocatalysis for non-CO2 greenhouse gases removal: an emerging negative emission technology)	non-CO2 greenhouse gases		Dec-20	4 years	€455 400.00	€276 000.00	PC	H2020-MSCA-RISE-2019	TRL > 3	https://cordis.europa.eu/project/id/871998
PolyNanoCat (Polymer Nanoparticle for Hydrogen Evolution)	Carbon Dioxide, Water	Hydrogen	Jan-21	2 years	€224 933.76	€224 933.76	PC	H2020-MSCA-IF-2019	TRL 1-3	EU H2020 Project "POLYNANOCAT (Polymer Nanoparticle for Hydrogen Evolution)": description, partecipants, costs and EC-fundings (fabiodisconzi.com)
USHPP (Unassisted photochemical water oxidation to solar hydrogen peroxide production)	Water	Hydrogen Peroxide	Jan-21	2 years	€224 933.76	€224 933.76	PC	H2020-MSCA-IF-2019	TRL > 3	Unassisted photochemical water oxidation to solar hydrogen peroxide production | USHPP Project | Fact Sheet | H2020 | CORDIS | European Commission (europa.eu)
PhotoReAct (Photocatalysis as a tool for synthetic organic chemistry)	Visible light	Fine Chemicals	Jan-21	4 years	€3 989 831.04	€3 989 831.04	PC	H2020-MSCA-ITN-2020	TRL > 3	https://cordis.europa.eu/project/id/956324
BioPhoCS (Bio- & Photo-Catalytic Methods for the Construction of Enantiomerically Pure C-S Bonds in Thiols and Sulphides)	Thioketones and Thioalkenes	Enantiomerically pure sulfur compounds	Jan-21	2 years	€224 933.76	€224 933.76	PC	H2020-MSCA-IF-2018	TRL 1-3	https://cordis.europa.eu/project/id/838326

Project	Starting molecule(s)	Target molecule(s)	Start date	Duration	Total Budget	EU contribution	Solar-driven Technology	Funding Call	TRL Scale	Project Website
Photo2Bio (Photo-Organocatalytic CO2 Valorisation into Bioactive Added-Value Molecules)	Carbon Dioxide	Amino acids, diverse complex molecular architectures	Feb-21	2 years	€183 473.28	€183 473.28	PC	H2020-MSCA-IF-2019	TRL 1-3	https://cordis.europa.eu/project/id/891908
CATCH (Cross-dimensional Activation of Two-Dimensional Semiconductors for Photocatalytic Heterojunctions)	Visible light	Hydrogen/water purification	May-21	5 years	€1 999 946.00	€1 999 946.00	PC	ERC-2020-COG	TRL 1-3	https://cordis.europa.eu/project/id/101002219
MicrobialLEAF (Cascade synthesis of ethanol and acetate via microbial fermentation of syngas produced photoelectrochemically by molecular catalysts on BiVO4-perovskite tandem artificial leaf)	Carbon Dioxide	Ethanol, Acetate	Jun-21	2 years	€224 933.76	€224 933.76	PEC	H2020-MSCA-IF-2019	TRL 1-3	Cascade synthesis of ethanol and acetate via microbial fermentation of syngas produced photoelectrochemically by molecular catalysts on BiVO4-perovskite tandem artificial leaf | MicrobialLEAF Project | Fact Sheet | H2020 | CORDIS | European Commission (europa.eu)
RELICS (REfining LIgnin by advanced Catalytic schemes powered by Sunlight)	Lignin	Phenolic aldehydes/ ketones	Jun-21	5 years	€1 536 183.00	€1 536 183.00	PC/PEC	ERC-2020-STG	TRL 1-3	https://cordis.europa.eu/project/id/948829
FENCES (Ferroelectric Nanocomposites for Enhanced Solar Energy Efficiency)	Carbon Dioxide, Water	Fuel	Jun-21	5 years	€1 999 903.00	€1 999 903.00	PC	ERC-2020-COG	TRL > 3	https://cordis.europa.eu/project/id/101001626
METHASOL (International cooperation for selective conversion of CO2 into METHAnol under SOLar light)	Carbon Dioxide	Methanol	Jul-21	3 years	€5 190 102.50	€3 999 633.75	PC	H2020-LC-SC3-2020-NZE-RES-CC	TRL > 3	International cooperation for selective conversion of CO2 into METHAnol under SOLar light | METHASOL Project | Fact Sheet | H2020 | CORDIS | European Commission (europa.eu)
NEFERTITI (Innovative photocatalysts integrated in flow photoreactor systems for direct CO2 and H2O conversion into solar fuels)	Carbon Dioxide	Ethanol/longer chain alcohols	Jul-21	4 years	€4 569 127.50	€3 844 427.50	PC	H2020-LC-SC3-2020-NZE-RES-CC	TRL > 3	http://projectnefertiti.eu/
MMOF4PPS (Unraveling the Photobehavior of Novel Mixed-metal MOFs for Efficient New Photocatalysts)	Light	Sustainable chemicals	Jul-21	2 years	€160 932.48	€160 932.48	PC	H2020-MSCA-IF-2020	TRL 1-3	https://cordis.europa.eu/project/id/101032472

Project	Starting molecule(s)	Target molecule(s)	Start date	Duration	Total Budget	EU contribution	Solar-driven Technology	Funding Call	TRL Scale	Project Website
CHARM (From CO2 to Hydrocarbons in A circular bioelectRo- and photo-cheMical system)	Biomass and CO2	Light weight hydrocarbons	Aug-21	2 years	€187 572.48	€187 572.48	PC	H2020-MSCA-IF-2019	TRL > 3	CHARM: From CO2 to Hydrocarbons in A circular bioelectRo- and photo-cheMical system | CHARM Project | Fact Sheet | H2020 | CORDIS | European Commission (europa.eu)
BIO-LIGHT (Photoexcitation for New-to-Nature Enzymatic Reactions)		Chiral molecules adorned with biologically relevant scaffolds	Aug-21	2 years	€160 932.48	€160 932.48	PC	H2020-MSCA-IF-2020	TRL > 3	https://cordis.europa.eu/project/id/101032077
PHOTOBIOCATH-CO2 (Photocathode engineering for efficient photobioelectrochemical CO2 reduction to formate)	Carbon Dioxide	Formate	Sep-21	2 years	€160 932.48	€160 932.48	PEC	H2020-MSCA-IF-2020	TRL 1-3	https://cordis.europa.eu/project/id/101024839
SolTIME (Solar Fuel Generation through Photoelectrochemical Reduction of CO2 Using Copper Porphyrins in Molecularly Designed Reaction Environments)	Carbon Dioxide, Water	Methane, Ethanol	Sep-21	2 years	€172 932.48	€172 932.48	PEC	H2020-MSCA-IF-2020	TRL 1-3	https://cordis.europa.eu/project/id/101031365
PhotoPhos (Photoinduced Decarboxylative Phosphorylations)	α-Amino carboxylic acids	α-Aminophosphonic acids	Sep-21	2 years	€224 933.76	€224 933.76	PC	H2020-MSCA-IF-2020	TRL 1-3	https://cordis.europa.eu/project/id/101027513
CONDOR (COmbined suN-Driven Oxidation and CO2 Reduction for renewable energy storage)	Carbon Dioxide	Hydrogen, CO, Methanol, DME	Nov-21	3 years	€4 087 866.25	€3 989 116.25	PEC	H2020-LC-SC3-2020-RES-RIA	TRL > 3	COmbined suN-Driven Oxidation and CO2 Reduction for renewable energy storage | CONDOR Project | Fact Sheet | H2020 | CORDIS | European Commission (europa.eu)
REPLY (REshaping Photocatalysis via Light-Matter hYbridization in Plasmonic Nanocavities)	Water	Hydrogen	Nov-21	5 years	€1 986 250.00	€1 986 250.00	PC	ERC-2020-COG	TRL 1-3	https://cordis.europa.eu/project/id/101002422
HyPhoCO (Organic/Inorganic Hybrid Photoelectrodes for sustainable CO2 reduction)	Carbon Dioxide	Value-added chemicals, fuels	Jan-22	2 years	€160 932.48	€160 932.48	PEC	H2020-MSCA-IF-2020	TRL 1-3	https://cordis.europa.eu/project/id/101030782
PLACABIN (Plasmon-Driven Catalysis on Bimetallic Nanostructures: Au-Pd for the Selective Oxygenation of Hydrocarbons)	Hydrocarbons	Oxygen-containing compounds	Jan-22	2 years	€224 933.76	€224 933.76	PC	H2020-MSCA-IF-2020	TRL 1-3	https://cordis.europa.eu/project/id/101022498
